# Liraglutide-Conjugated Poly(methyl vinyl ether-*alt*-maleic acid)-Coated Core–Shell Upconversion Nanoparticles for Theranostics of Diabetes

**DOI:** 10.1021/acsami.5c11275

**Published:** 2025-07-16

**Authors:** Oleksandr Shapoval, Hana Engstová, Miroslav Šlouf, Olga Kočková, Andrea Dlasková, Martin Jabůrek, Aminadav Halili, Alexandra Mozheitová, Daniel Jirák, Petr Ježek, Daniel Horák

**Affiliations:** † Institute of Macromolecular Chemistry of the Czech Academy of Sciences, Heyrovského nám. 2, 162 00 Prague 6, Czech Republic; ‡ Institute of Physiology of the Czech Academy of Sciences, Vídeňská 1083, 142 20 Prague 4, Czech Republic; § Institute for Clinical and Experimental Medicine, Vídeňská 1958/9, 140 21 Prague 4, Czech Republic; ∥ Institute of Biophysics and Informatics, First Faculty of Medicine, Charles University, 121 08 Prague 1, Czech Republic; ⊥ Faculty of Health Studies, Technical University of Liberec, 461 17 Liberec, Czech Republic

**Keywords:** upconversion, nanoparticles, liraglutide, theranostics, diabetes, poly(methyl vinyl ether-alt-maleic acid), Flamma

## Abstract

In the diagnostics of diabetes, specific targeting of drugs (e.g., liraglutide) to insulin-deficient β-cells with their simultaneous noninvasive imaging is currently needed. In this report, liraglutide (LGL)-conjugated poly­(methyl vinyl ether-*alt*-maleic acid) (PMVEMA)-coated core–shell NaYF_4_:Yb,Er,Fe@NaYF_4_:Nd upconversion nanoparticles (CS-UCNPs) have been developed, thoroughly physicochemically characterized, and evaluated *in vivo*. Novel codoping of Fe^2+^, Yb^3+^, and Er^3+^ ions in the host NaYF_4_ induced upconversion emission in the red region at both 980 and 808 nm excitation, making the particles suitable for deep-tissue imaging. Surface functionalization with PMVEMA provided colloidal stability and facilitated covalent conjugation with LGL, enabling targeted binding to GLP-1 receptors on pancreatic β-cells, increasing glucose-stimulated insulin secretion from isolated Langerhans islets. Biocompatibility of CS-UCNP@PMVEMA-LGL nanoparticles was confirmed by the trypan blue dye exclusion assay. When the fluorescent dye Flamma was conjugated to the nanoparticles, *in vivo* fluorescence imaging revealed significantly enhanced accumulation of CS-UCNP@PMVEMA-LGL-Flamma nanoparticles in the pancreas 24 h after intramuscular injection compared with intravenous administration, with luminescence intensity approximately doubled. The improved pancreatic targeting efficiency was attributed to enhanced binding to GLP-1 receptors. Confocal microscopy and elemental analysis confirmed receptor-mediated uptake of the nanoparticles by internalization and their localization within pancreatic β-cells. These findings highlight the potential of CS-UCNP@PMVEMA-LGL nanoparticles as biocompatible targetable imaging agents with future applications in pancreatic diagnostics.

## Graphical abstract

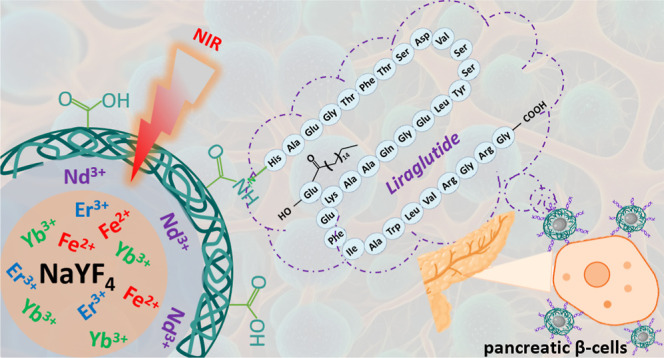

## Introduction

Diabetes mellitus is a pandemic chronic metabolic disease that is often fatal and incurable in its progressive stages. In type 1 diabetes, insulin production is completely stopped due to an autoimmune attack on the pancreatic β-cells. In contrast, in the more common type 2 diabetes acquired in adulthood and old age, main manifestation is insulin resistance accompanied by a gradual reduction of β-cell mass and impairment of β-cell function.^1^ Hyperinsulinemia in the initial stages is followed by poor insulin secretion. With the developed insulin resistance, despite insulin secretion by pancreatic islet β-cells, the insulin receptor pathway enabling glucose uptake in peripheral tissues is impaired. In advanced stages, β-cell dedifferentiation or transdifferentiation occurs, causing decline of β-cell mass.^2,3^ The recent introduction of new insulin and incretin analogues has revolutionized diabetes treatment because incretins, besides amplification of insulin secretion, provide trophic effects on β-cells. Similarly, noninvasive glycemic monitoring systems have made life of diabetic patients more comfortable. Despite these efforts, most people with type 1 diabetes find it difficult to achieve and maintain consistent glycemic control.^2–4^ Last but not least, new and safe treatments should be also explored, as diabetes is associated with the risk of cardiovascular and other complications.^5^ An example of a new treatment for type 2 diabetes in recent years is the introduction of liraglutide (LGL), a glucagon-like peptide-1 receptor agonist. Slight modification of native GLP-1 enabled up to 24 h efficacy, which was further extended to a week with semaglutide due to its effective binding to albumin. These incretin analogues amplify insulin release from pancreatic islet β-cells, improving their function and preserving their identity. At the same time, they also reduce excessive glucagon release, thereby decreasing the risk of hypoglycemia, obesity, and cardiovascular disorders.^6,7^ LGL was approved by the US Food and Drug Administration in 2014 as a weight loss medication for obese or overweight adults and in 2019 for the treatment of children aged 10 years and older with type 2 diabetes. However, long-term oral or subcutaneous administration of antidiabetic drugs might be associated with adverse effects on the kidneys, liver, or the macrovascular system.

Recent advances in the development of novel nanomaterials have enabled new ways to detect diabetic biomarkers and treat diabetes using biocompatible drug-carrying silica-coated nanoparticles for oral administration, improving the comfort for patients. Due to their small size, nanoparticle systems can deliver poorly soluble bioactive molecules into circulation and release antidiabetic drugs such as insulin,^8^ metformin,^9^ exenatide,^10^ glimepiride,^11^ LGL,^12^ etc., in a controlled manner, leading to higher efficacy and fewer side effects.^13,14^ For these purposes, upconversion nanoparticles (UCNPs) based on rare earths are being tested, which not only transport the drug but also enable precise detection of its movement in the body. UCNPs that are capable of converting near-infrared excitation into UV/visible emissions consist of a host matrix (mainly NaYF_4_ or NaGdF_4_) and dopants, i.e., an energy-providing sensitizer (Yb^3+^) and a visible light-emitting activator (Er^3+^, Tm^3+^, Ho^3+^, Tb^3+^, etc.).^15^ Unlike conventional fluorescent labels, the advantages of UCNPs are a sharp emission bandwidth, low cytotoxicity, high chemical stability, deep penetration of NIR light into tissues, long lifetime, and a high signal-to-noise ratio.^16^ To achieve increased luminescence efficiency, UCNPs can be modified to core–shell structures with two or more lanthanide activators in the core and a NaYF_4_ or NaGdF_4_ shell that eliminates detrimental cross-relaxation.^17^

For use in living organisms, UCNPs need to be surface-modified so that they are colloidally stable as the aggregation in biological fluids leads to functional failure. Polymers are mainly used for this purpose, e.g., modification with carboxyl-functionalized 3,4-dihydrocinnamic acid or poly­(monoacryloxyethyl phosphate) with a negative surface charge resulted in a better uniform distribution of particles in biological buffers than amino-functionalized UCNPs coated with (aminomethyl)­phosphonic acid or (3-aminopropyl)­triethoxysilane with a positive surface charge.^18^ Polymers with multiple anchoring negatively charged phosphate groups, such as poly­(oligo­(ethylene glycol) methyl ether acrylate)-*block*-poly­(monoacryloxyethyl phosphate), provided excellent colloidal stability for UCNPs in physiologically relevant buffers.^19^ Poly­(methyl vinyl ether-*co*-maleic acid)-coated UCNPs were found to be well colloidally stable in phosphate buffer saline (PBS) and Dulbecco’s modified Eagle’s medium (DMEM).^20^ UCNPs protected by poly­(acrylic acid), polyallylamine, or poly­(ethylene glycol) showed long-term colloidal stability in low-concentration aqueous dispersions.^21^ In addition, UCNPs with coatings consisting of a poly­(isobutylene-*alt*-maleic anhydride) backbone functionalized with phosphonate groups and poly­(ethylene glycol) moieties exhibited high long-term colloidal stability in biologically relevant buffers.^22^ Other examples of polymers providing colloidal stability to UCNPs are polyvinylpyrrolidone^23^ and polyethylenimine.^24^

The aim of this report was to design novel poly­(methyl vinyl ether-*alt*-maleic acid) (PMVEMA)-coated core–shell NaYF_4_:Yb,Er,Fe@NaYF_4_:Nd UCNPs conjugated with LGL and test their retention in the pancreas *in vivo* due to binding to the GLP-1 receptors (GLP1R), which are enumerated in β-cells. At the same time, the differences in efficiency between intravenous and intramuscular administration and the ability to visualize β-cells of mouse pancreatic islets *in vivo* were studied. Based on the biodistribution properties, the designed nanoparticles could be useful for future diagnostics of β-cell mass or special theranostics of diabetes and related pharmacokinetic research and applications.

## Experimental Section

### Materials

Chlorides of yttrium (YCl_3_; 99%), erbium (ErCl_3_× 6 H_2_O; 99%), ytterbium (YbCl_3_; 99%), neodymium­(NdCl_3_; 99%), iron (FeCl_2_ × 4 H_2_O; 99%), 2-(*N*-morpholino)­ethanesulfonic acid (MES), *N*-(3-(dimethylamino)­propyl)-*N*′-ethylcarbodiimide (EDC), *N*-hydroxysulfosuccinimide sodium salt (sulfo-NHS), octadec-1-ene (90%), and PBS were purchased from Sigma-Aldrich (St. Louis, MO, USA). DMEM and RMPI 1640 medium were purchased from the Institute of Molecular Genetics (Prague, Czech Republic). Hexane (99.5%), methanol (99.5%), dimethyl sulfoxide (DMSO; 99.99%), sodium hydroxide, sodium chloride, and oleic acid (OA) were obtained from Lach-Ner (Neratovice, Czech Republic). Nitric acid (65–69%; Analpure Ultra) was purchased from Analytika (Prague, Czech Republic). PMVEMA (*M*_w_ = 60 kDa; PMVEMA; Figure 1a) was obtained from Scientific Polymer Products (Ontario, NY, USA). A commercial LGL pen (Saxenda; Figure 1b) was provided as an injection by the Thomayer Hospital Pharmacy (Prague, Czech Republic). Flamma 749 hydrazide (briefly named Flamma) was purchased from BioActs (Incheon, Korea). All other chemicals were from commercial sources and used without further purification. Buffers and solutions were prepared from ultrapure water obtained by reverse osmosis with UV treatment (Milli-Q IQ7000 system; Merck; Darmstadt, Germany).

**Figure 1:**
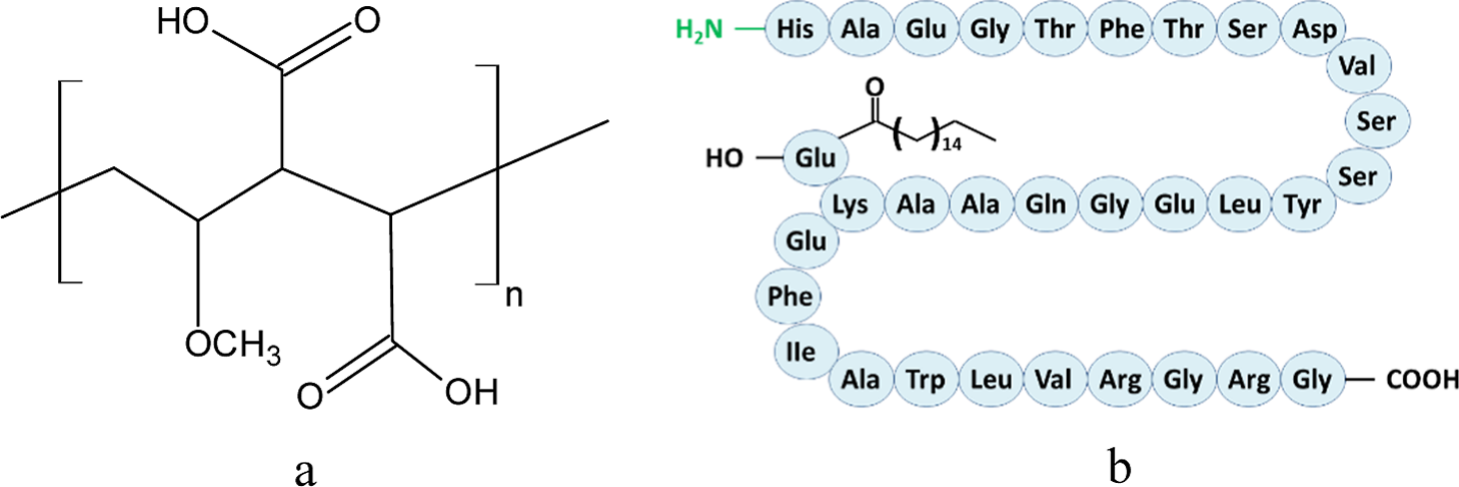
Chemical structure of (a) PMVEMA and (b) LGL.

### Preparation of C- and CS-UCNPs and Their Surface Modification with PMVEMA

Core NaYF_4_:Yb,Er,Fe nanoparticles (C-UCNPs) and core–shell NaYF_4_:Yb,Er,Fe@NaYF_4_:Nd nanoparticles (CS-UCNPs) were prepared according to previously published reports.^25,26^ Briefly, yttrium­(III), ytterbium­(III), erbium­(III), and iron­(II) chlorides (0.6/0.2/0.15/0.05 mol/mol/mol/mol, respectively) and oleic acid (6 mL) were dissolved in octadec-1-ene (15 mL) at 170 °C for 30 min under an Ar atmosphere. The mixture was cooled down to room temperature (RT), a methanolic solution of NaOH (2.5 mmol) and NH_4_F (4 mmol) was dropwise added, and the mixture was slowly heated to 120 °C under argon. The heating continued at 300 °C for 1.5 h with stirring under an Ar atmosphere until methanol evaporated. The resulting NaYF_4_:Yb,Er,Fe C-UCNPs were separated by centrifugation (3460 rcf) for 30 min and washed with hexane/ethanol mixture (1/4 v/v) four times.

The core–shell NaYF_4_:Yb,Er,Fe@NaYF_4_:Nd nanoparticles (CS-UCNPs) were prepared according to the same procedure as described above using YCl_3_ (0.4 mmol), NdCl_3_ (0.1 mmol), and oleic acid (6 mL) dissolved in octadec-1-ene (15 mL). The mixture was heated at 160 °C for 30 min under an Ar atmosphere and cooled to RT, and hexane dispersion (15 mL) of NaYF_4_:Yb,Er,Fe nanoparticles (150 mg) and methanolic solution of NaOH (1.25 mmol) and NH_4_F (2 mmol) were added. Methanol and hexane were evaporated at 70 °C and the mixture was heated at 300 °C for 1.5 h under an Ar atmosphere. The CS-UCNPs were separated by centrifugation (3460 rcf) for 30 min and washed in hexane/ethanol, ethanol, ethanol/water, and finally water. For physicochemical characterization, part of the dispersion was vacuum-dried at RT for 3 days.

PMVEMA-coated CS-UCNPs (CS-UCNP@PMVEMA) were prepared according to a previously described procedure.^27^ Briefly, PMVEMA (500 mg) was dissolved in water (15 mL; pH = 7.4 was adjusted by adding 2 M NaOH) at RT. An aqueous dispersion (2 mL) of CS-UCNPs (10 mg) was added dropwise to the PMVEMA solution with shaking at RT for 30 min, and the mixture was stirred at 70 °C for 16 h. The resulting CS-UCNP@PMVEMA nanoparticles were washed three times with water using centrifugation (14,100 rcf) for 20 min to remove unbound PMVEMA and redispersed in water.

### Conjugation of LGL on CS-UCNP@PMVEMA Nanoparticles

Prior to conjugation, 1 mL of commercial LGL (6 mg LGL/ml) was desalted and transferred to sodium borate buffer (0.05 M; pH = 10.6) by centrifugation (3460 rcf) using a Vivaspin 2 centrifugal concentrator (MWCO = 3000 Da; Vivaproducts; Littleton, MA, USA). For covalent conjugation, the carboxyl groups of PMVEMA were reacted with the terminal amino groups of LGL using EDC/NHS coupling chemistry. The aqueous CS-UCNP@PMVEMA dispersion (2 mg/mL; 1 mL) was centrifuged (14,100 rcf) for 30 min, the supernatant was removed, and the particles were washed twice with MES buffer (pH = 5.2) and redispersed in it (2 mL) to a concentration of 2 mg/mL. 100 μL of a mixture of EDC (1 mg) and sulfo-NHS (2.5 mg) in MES buffer (200 μL) was added to 1 mL of the CS-UCNP@PMVEMA dispersion (2 mg/mL) and the mixture was incubated for 2 h under shaking. The particles were purified from excess EDC and sulfo-NHS by centrifugation (14,100 rcf) for 20 min and dispersed in MES buffer. Activated particles (1 mL; 2 mg/mL) were added dropwise to a solution of LGL (1 mL; 6 mg/mL) in 0.05 M sodium borate buffer (pH = 10.6) and the mixture was stirred at RT for 24 h. To ensure complete conjugation of LGL to the particles, an excess of LGL was added to the reaction, the pH of which was maintained between 9.6 and 10. Finally, the conjugate denoted as CS-UCNP@PMVEMA-LGL was separated on a poly­(ether sulfone) filter (VWR International; Prague, Czech Republic; MWCO = 30,000 Da) by centrifugation (14,000 rcf) for 20 min, washed several times with water, and redispersed to the desired concentration. The supernatant was collected and analyzed by UV–vis spectroscopy to determine the conjugation efficiency. Optionally, the unwashed CS-UCNP@PMVEMA-LGL particle dispersion was recovered and used for conjugation with Flamma.

### Conjugation of Flamma 749 Hydrazide to CS-UCNP@PMVEMA-LGL Nanoparticles

A solution of Flamma 749 hydrazide (Supporting Information; Figure S1; 1 mg) in DMSO (0.5 mL) was dropwise added to an unwashed CS-UCNP@PMVEMA-LGL dispersion (2 mL; 2 mg/mL), and the mixture was magnetically stirred at RT for 24 h in the dark. Unbound Flamma was removed using a centrifugation poly­(ether sulfone) filter (MWCO = 30,000 Da) and the resulting CS-UCNP@PMVEMA-LGL-Flamma nanoparticles were redispersed in water to the desired concentration.

### Characterization of the Particles

The particle morphology, elemental composition, and crystal structure were examined using a Tecnai Spirit G2 transmission electron microscope (TEM; FEI; Brno, Czech Republic).^27^ The number-average diameter (*D*_n_ = Σ*N*_*i*_ *D*_*i*_/Σ*N*_*i*_), weight-average diameter (*D*_w_ = Σ*N*_*i*_ *D*_*i*_^4^/Σ*N*_*i*_ *D*_*i*_^3^), and dispersity (*D̵* = *D*_w_/*D*_n_) were calculated by measuring at least 300 nanoparticles from four random TEM/bright field (TEM/BF) micrographs using ImageJ software v. 1.52p (National Institutes of Health; Bethesda, MD, USA);^28^ *N*_*i*_ is the number and *D*_*i*_ is the diameter of the *i*-th particle. A TEM microscope was equipped with an energy-dispersive X-ray (EDX) spectrometer (Mahwah, NJ, USA) for analysis of the elemental composition of the nanoparticles. The crystal structure was verified by the selected area electron diffraction (TEM/SAED) patterns, which were compared with the theoretically calculated powder X-ray diffraction patterns (PXRD) of cubic and hexagonal phases of NaYF_4_. The processing of the TEM/SAED patterns and the calculations of PXRD were performed with open source package EDIFF.^29^

Hydrodynamic nanoparticle diameter (*D*_h_), polydispersity (*PD*), and ξ-potential were measured using dynamic light scattering (DLS; ZSU 5700 Zetasizer Ultra Instrument; Malvern Instruments; Malvern, UK) at 25 °C; *D*_h_ and *PD* were calculated from the intensity-weighted distribution function obtained by CONTIN analysis of the correlation function embedded in Malvern software. Temperature-dependent changes in particle weight in air were measured with a PerkinElmer TGA 7 thermogravimetric analyzer (Norwalk, CT, USA) over a temperature range of 30–800 °C at a constant heating rate of 10 °C/min.

Samples for elemental analysis were digested with HNO_3_ (1 mL) in a Biotage initiator microwave reactor (Uppsala, Sweden) and the Y^3+^, Er^3+^, and Yb^3+^ concentrations were quantified using a NexION 2000B inductively coupled plasma mass spectrometer (ICP–MS; PerkinElmer; Waltham, MA, USA). Standard rare earth metal solutions (100 mg/L) in 5% HNO_3_ diluted to a concentration of 0.002–0.18 μg/L were used to obtain the calibration curve. The metal ions in the samples were stabilized with 2.5% HNO_3_. The Fe^2+^ content was determined on a PerkinElmer 3110 atomic absorption spectrometer (AAS; PerkinElmer).

Emission and excitation spectra were recorded in a Hellma 114F-QS cuvette (10 × 4 mm path length; Sigma-Aldrich) at RT on an FS5 Edinburgh Instruments spectrofluorometer (Edinburgh, UK) equipped with continuous (150 W) and pulsed xenon lamps and coupled with CW 808 (MDL-III-808) and CW 980 (MDL-III-980) infrared diode lasers as an excitation source with a nominal laser power of 2 W (beam size of 5 × 8 mm^2^). A 750FL07-50S cutoff short-pass filter with a wavelength of 750 nm (Andover Corporation; Salem, NH, USA) was used to minimize emission from scattering of the 808 nm excitation laser.

Conjugation of LGL to the particle surface was confirmed by biuret colorimetric assay detecting amide bonds in solution^30^ and fluorescence spectroscopy (FS5 Edinburgh Instruments). The biuret test was performed by adding 2 drops of 2 M NaOH and 2 drops of 1 wt % aqueous CuSO_4_ × 5H_2_O solution to the aqueous dispersion of CS-UCNP@PMVEMA-LGL particles (0.5 mL; 1 mg/mL), which caused the solution to become colored. The excitation spectra of CS-UCNP@PMVEMA nanoparticles before and after conjugation of LGL were measured at 297 nm (λ_em_ = 490 nm) on an FS5 Edinburgh Instruments spectrofluorometer.

LGL conjugated to CS-UCNP@PMVEMA nanoparticles was quantified by measuring the absorbance of the supernatant before and after LGL conjugation using a Specord 250 Plus UV–vis spectrophotometer (Analytik; Jena, Germany) at 290 nm. The calibration curve was obtained from the absorption spectra of desalted LGL at different concentrations (0.06–1.5 mg/mL) in borate buffer (pH = 10.6; Figure S2a,b). The amount of bound LGL to particles was calculated as the difference between its initial amount and the amount of unbound peptide.^31^

### Biological Experiments

The biological experiments were ethically reviewed and approved under European Directive 86/609/EEC by the Czech Central Commission for Animal Welfare, the Ethics Committee of the First Faculty of Medicine, Charles University, and the Ministry of Education, Youth, and Sports of Czech Republic. All procedures adhered to Act No. 246/1992 Coll. on the protection of animals against cruelty and Decree No. 419/2012 on the protection of experimental animals, in compliance with the regulations of the European Parliament.

### Pancreatic Islet Isolation

Two male and two female mice of the C57Bl/6J strain (The Jackson Laboratory; Bar Harbor, MN, USA) were anesthetized using a mixture of Zoletil (SG-VET; Kopřivnice, Czech Republic) and 2% Rometar (Bioveta; Ivanovice na Hané, Czech Republic). Pancreases were digested with collagenase and 150–200 pancreatic islets per mouse were isolated by centrifugation on a Ficoll density gradient.^32^

### Cytotoxicity of CS-UCNP@PMVEMA-LGL Nanoparticles

The trypan blue exclusion test (Thermo Fisher Scientific) was used to determine the cytotoxicity of CS-UCNP@PMVEMA-LGL nanoparticles on INS-1 cells (AddexBio; San Diego, CA, USA; cat. no. C0018009) derived from X-ray-induced transplantable rat insulinoma cells. The cells were cultured in RMPI 1640 medium with 11 mM glucose at 37 °C for 48 h in a humidified atmosphere with 5% CO_2_ and then incubated with nanoparticles (0.01–0.4 mg/mL) for 2 and 24 h. *In vitro* cell viability was determined by 0.4% trypan blue staining, and the fraction of living cells was counted on a Luna II automated cell counter (Logos Biosystems; Gyeonggi-do, South Korea).

### Determination of Insulin Secretion Rate by Islet Perifusion

Dynamic insulin release from islets was determined by perifusion, where ∼100 islets were placed on an Econo size-exclusion chromatography column (1 × 7 cm) packed with Bio-Gel P4 beads and equipped with a flow adapter (Bio-Rad; Hercules, CA, USA).^32^ Pancreatic islets were washed for 60 min in a continuous flow of glucose-free Krebs–Ringer HEPES (KRH) buffer (135 mmol/L NaCl, 3.6 mmol/L KCl, 10 mmol/L HEPES, 0.5 mmol/L MgCl_2_, 1.5 mmol/L CaCl_2_, 0.5 mmol/L NaH_2_PO_4_ and 0.1% BSA; pH = 7.4) with basal 2.5 mM glucose. Then, KRH buffer with insulin-stimulating 25 mM glucose was added to the KRH perifusion medium and either without addition or with the addition of Exendin-4 (GLP-1 analogue) or CS-UCNP@PMVEMA-LGL nanoparticles (200 μL; 5 mg/mL); the perfusate was collected for 60 min at a rate of 0.4 ± 0.1 mL/min. To release the maximum insulin, 30 mM KCl was added 45 min after glucose introduction.

Insulin was detected with a highly sensitive mouse insulin ELISA kit (BioVendor; Brno, Czech Republic). To isolate DNA, islets were lysed with a buffer containing 10 mM Tris-HCl (pH = 7.5), 150 mM NaCl, 5 mM ethylenediaminetetraacetic acid (EDTA; pH = 8.0), and 0.5% sodium dodecyl sulfate, supplemented with 50 μg/mL proteinase K. After incubation at 55 °C overnight, the lysates were collected and centrifuged (12,000*g*) at RT for 10 min. The supernatants were treated with an equal volume of isopropanol, incubated for 10 min, and centrifuged at 12,000 *g* for 10 min. The resulting pellets were dissolved in 10 mM Tris–EDTA (pH = 7.4) at 55 °C for 1 h, and the DNA concentration was measured using a Quant-iT PicoGreen dsDNA assay (Invitrogen; Waltham, MA, USA).

### Confocal Microscopy of CS-UCNP@PMVEMA-LGL-Labeled INS-1E Cells and Pancreas

The interaction of nanoparticles with cells was tested on INS-1E cells serving as a model of pancreatic β-cells because they respond to glucose and secrete insulin.^32^ The cells were cultured in 11 mM glucose and RPMI 1640 medium supplemented with 5% (v/v) fetal calf serum, 10 mM HEPES, 1 mM pyruvate, 50 mM mercaptoethanol, 50 IU/ml penicillin, and 50 mg/mL streptomycin and seeded on coverslips. After incubation with CS-UCNP@PMVEMA-LGL nanoparticles (0.3–0.4 mg/mL) for 0–24 h, the cells were viewed with a Leica SP8 laser confocal microscope (Wetzlar, Germany) with excitation at 808 and 980 nm using a Coherent 200 fs pulsed Chameleon laser with a power of 160 and 100 mV, respectively.

CS-UCNP@PMVEMA-LGL particle dispersions were also intramuscularly and/or intravenously (in the tail) administrated in C57Bl/6J mice. After 15 min or 24 h, the mice were anesthetized (see above), and pancreases were excised, placed on coverslips, and viewed by a Leica SP8 laser confocal microscope at 808 and 980 nm with pulsed 200 fs excitation.

### Quantification of Rare Earth Ions in the Pancreas by ICP–MS Analysis

In order to remove water and obtain powder, freshly excised pancreas after intramuscular and/or intravenous administration of the particles in C57Bl/6J mice was vacuum freeze-dried for 48 h on an L4-110 PRO lyophilizer (Gregor Instruments; Sázava, Czech Republic). Concentrations of Y^3+^ and Yb^3+^ ions in powdered pancreas (20–40 mg) were determined by ICP–MS as described above.

### *In Vivo* Optical Imaging of Mouse Organs

To evaluate the biodistribution of CS-UCNP@PMVEMA-LGL-Flamma nanoparticles in target organs, *in vivo* fluorescence imaging was performed using two female nu/nu nude mice (Hsd: athymic Nude-Fox n1nu; AnLab; Prague, Czech Republic) and two female C57B1/6J black mice (MANLAB IKEM; Prague, Czech Republic), each weighing between 18 and 22 g. The animals were housed in laminar flow cabinets with radiation-sterilized SAWI bedding (Jelu-Werk; Rosenberg, Germany), fed an irradiated diet (Ssniff Spezialdiäten; Soest, Germany), and had ad libitum access to autoclaved water. Biodistribution of UCNP@PMVEMA-LGL-Flamma nanoparticles in target organs was assessed at multiple time points (10 min, 1, 3, and 24 h) postinjection. Based on preliminary imaging results, the 3 and 24 h time points were selected for detailed analysis due to their pronounced and anatomically distinguishable fluorescence signals.

Nanoparticles were administered either intramuscularly (outer thigh) or intravenously (tail vein). *In vivo* imaging was conducted using a Spectral Instruments Imaging system (Bruker; Tucson, AZ, USA) with excitation/emission at 675/770 nm for all measurements, except for the 3 h intravenous administration in C57Bl/6J mice (605/770 nm) to optimize signal acquisition. The imaging of dissected organs was performed with excitation at 675 nm and emission at 730 nm. Black mice were used for the 3 h time point, and nude mice were used for the 24 h time point. Fluorescence images were analyzed using Aura software (Spectral Instruments Imaging) and luminescence was quantified as photons/s/cm^2^.

### Transmission Electron Microscopy of Pancreatic Cells and Islets

The islets and cells were fixed for 24 h in 2.5% glutaraldehyde in 0.1 M cacodylate buffer (pH = 7.2) followed by 2% OsO_4_ staining in the same buffer. To visualize the conspicuous mitochondrial membranes, postfixation with 2% of OsO_4_ and 0.8% of K_4_[Fe­(CN)_6_] in PBS buffer was performed. Fixed samples were dehydrated with an ascending series of ethanol and acetone and embedded in an Araldite-Poly/Bed 812 mixture (Polysciences; Warrington, PA, USA). Thin sections were cut on a Reichert-Jung Ultracut E ultramicrotome (Depew, NY, USA) and stained with uranyl acetate and lead citrate. Sections were examined and photographed using a JEOL JEM-1011 electron microscope. Fine structure measurements were performed using a Veleta camera and iTEM 5.1 software (Olympus Soft Imaging Solution; Tokyo, Japan).

## Results and Discussion

### C- and CS-UCNPs

In this report, core (C-UCNPs) and core–shell upconversion nanoparticles (CS-UCNPs) were prepared by high-temperature (300 °C) coprecipitation of rare earth and iron chlorides in octadec-1-ene as solvent in the presence of oleic acid as a stabilizer. The core consisted of NaYF_4_:Yb,Er,Fe crystal; the incorporation of transition Fe^2+^ ion (Mn^2+^ are also possible) into the core was aimed to increase the intensity of upconversion emission in the red region with negligible damage to living tissue and minimal background autofluorescence, which is important for *in vivo* imaging in clinical diagnostics.^33,34^ The shell around the NaYF_4_:Yb,Er,Fe core contained NaYF_4_:Nd, where Nd^3+^ was another sensitizer, enhancing the brightness and enabling excitation at 808 nm in the transparent NIR optical window with deep penetration into biological tissues.

While C-UCNPs were spherical in shape with a diameter of 34 nm (Figure S3a; Table 1), CS-UCNPs were typically cylindrical in shape with a length of 43 nm and a width of 35 nm (Figure S3d; Table 1), i.e., they were somewhat larger than the starting core nanoparticles, which is in agreement with previously published results.^27,35^ Both C- and CS-UCNPs were uniform in size (*D̵* = 1.01; Table 1), which is critical for the achievement of consistent physical and biological properties and reproducible results. TEM/SAED diffraction patterns of both C-UNCPs (Figure S3b) and CS-UCNPs (Figure S3e) are shown in the Supporting Information documenting that the crystal structure did not change during the final epitaxial growth. Finally, the TEM/EDX spectrum of the resulting CS-UCNPs confirmed the expected elemental composition (Figure S3g). The dominant peaks corresponded to the main components of sodium yttrium fluoride (Na, Y, and F) and to the carbon-coated copper support grid (C and Cu) on which the nanoparticles were deposited. In contrast, peaks with lower intensity corresponded to other ions in the NaYF_4_ matrix (Yb, Er, Nd); the iron peak could not be detected due to very low concentration of Fe^2+^ ions. However, the doping of NaYF_4_ with Fe^2+^ ions was confirmed by AAS; the iron concentration was 3 mol % (Table S1). Furthermore, the molar ratio of rare earth ion concentrations determined in C- and CS-UCNPs by ICP–MS corresponded well with the stoichiometric ratios used in the reaction mixture (Table S1).

**Table 1:** Characterization of the Nanoparticles^a^

particles	*D*_n_ (nm)	*D̵*	*D*_h_ (nm)	*PD*	ζ-potential (mV)
C-UCNPs	34	1.01	149 ± 3	0.15	30 ± 2
CS-UCNPs	43^b^–35^c^	1.01	175 ± 4	0.12	32 ± 3
CS-UCNP@PMVEMA	43^b^–35^c^	1.01	182 ± 2	0.17	–24 ± 1
CS-UCNP@PMVEMA-LGL	43^b^–35^c^	1.01	258 ± 5	0.18	–28 ± 1
CS-UCNP@PMVEMA-LGL-Flamma	43^b^–35^c^	1.01	264 ± 3	0.19	–36 ± 2

a C-UCNPs (NaYF_4_:Yb,Er,Fe) and CS-UCNPs (NaYF_4_:Yb,Er,Fe@NaYF_4_:Nd)—core and core–shell upconverting nanoparticles; PMVEMA—poly­(methyl vinyl ether-*alt*-maleic acid); LGL—liraglutide; *D*_n_—number-average diameter (TEM), *D̵*—dispersity (TEM), *D*_h_—hydrodynamic diameter (DLS), *PD*—polydispersity (DLS).

b Length.

c Width.

The hydrodynamic diameters (*D*_h_) of C- and CS-UCNPs in water measured by DLS reached 149 and 175 nm, respectively; the polydispersity of the particles was small (*PD* ≤ 0.15; Table 1), which was consistent with the TEM results. A larger hydrodynamic diameter than the number-average particle size according to TEM may indicate a slight tendency toward aggregation typical of uncoated particles. Both types of nanoparticles exhibited positive ζ-potentials of 30 and 32 mV, respectively, due to positively charged metal ions on the surface (Table 1).

Furthermore, the upconversion luminescence emission of C- and CS-UCNPs was measured under NIR excitation at 808 and 980 nm (Figure 2). It is worth mentioning that both increasing the Er^3+^ concentration to 15 mol.% and incorporating Fe^2+^ ions (5 mol.%) into the particles increased the intensity of upconversion luminescence in the red region, as shown in our previous paper.^36^ Nevertheless, to the best of our knowledge, there are so far no reports on upconversion luminescence of Fe^2+^-doped C-UCNPs under 808 nm excitation. While the commonly used NaYF_4_:Yb­(20 mol.%),Er­(2 mol.%) nanoparticles did not exhibit upconversion emission under the 808 nm excitation, the incorporation of Fe^2+^ into the core NaYF_4_:Yb­(20 mol.%),Er­(15 mol.%) particles resulted in upconversion luminescence, making possible excitation deep in the tissue (Figure 2a). Characteristic emission peaks originating from ^2^H_9/2_ → ^4^I_15/2_ (408 nm), ^2^H_11/2_ → ^4^I_15/2_ (522 nm), ^4^S_3/2_ → ^4^I_15/2_ (540 nm) and ^4^F_9/2_ → ^4^I_15/2_ (655 nm) transitions of Er^3+^ ions were observed in the spectrum of the C-UCNPs. Moreover, the presence of Fe^2+^ ions in C-UCNPs caused strong upconversion luminescence at 734 nm originating from the ^4^F_7/2_ → ^4^I_11/2_ transition of Er^3+^. Compared to the C-UCNPs, the introduction of the NaYF_4_:Nd shell around the C-UCNPs increased the intensity of upconversion emission at 408, 522–540, 655, and 734 nm by 45, 5, 7, and 1.5×, respectively, making the upconversion emission in the red region dominant after 808 nm excitation. The emission spectra of C-UCNPs excited at 980 nm showed characteristic Er^3+^ emission bands at 408 nm (^2^H_9/2_ → ^4^I_15/2_), 522 nm (^2^H_11/2_ → ^4^I_15/2_), 540 nm (^4^S_3/2_ → ^4^I_15/2_), 654 nm (^4^F_9/2_ → ^4^I_15/2_) and 806 nm (^4^I_9/2_ → ^4^I_15/2_; Figure 2b). As expected, compared to the C-UCNPs, introduction of the NaYF_4_:Nd shell demonstrated a 4 times higher upconversion intensity for both green and red emissions at 980 nm excitation. The increased upconversion luminescence could be assigned to the incorporation of Fe^2+^ ions into the cores and not to the involvement of the energy levels (d orbitals) of Fe^2+^ in the upconversion process, i.e. their direct influence on the f–f transitions of Er^3+^ and Yb^3+^. In addition, structural alterations in the host lattice induced by the replacement of larger lanthanide ions by Fe^2+^ ions with smaller ionic radii contributed to the increased luminescence.^37,38^ This led to the formation of intermediate energy states facilitating energy transfer between the Yb^3+^ sensitizer ions and the Er^3+^ activator ions, increasing the probability of forbidden f–f transitions in Er^3+^ and Yb^3+^, and providing efficient energy migration. It is then known from the literature that doping with transition metal ions (Cr^3+^, Fe^2+^, Fe^3+^, Mn^2+^ and Zn^2+^) reduced the nonradiative surface quenching by passivating the surface defects and induced imbalanced charges in the crystal lattices, thus improving luminescence efficiency.^39–41^ Especially, Fe^2+^ (5 mol.%)- and Fe^3+^ (20 mol.%)-doped NaYF_4_:Yb^3+^,Er^3+^ nanoparticles showed an eight- and seven-fold enhancement in red upconversion emission at 980 nm excitation, respectively.^42,43^ However, the mechanism for the enhanced Er^3+^ upconversion emissions by doping Fe^2+^ ions requires further investigation because iron ions have abundant energy levels, which occasionally leads to quenching of visible emission.^44^

**Figure 2:**
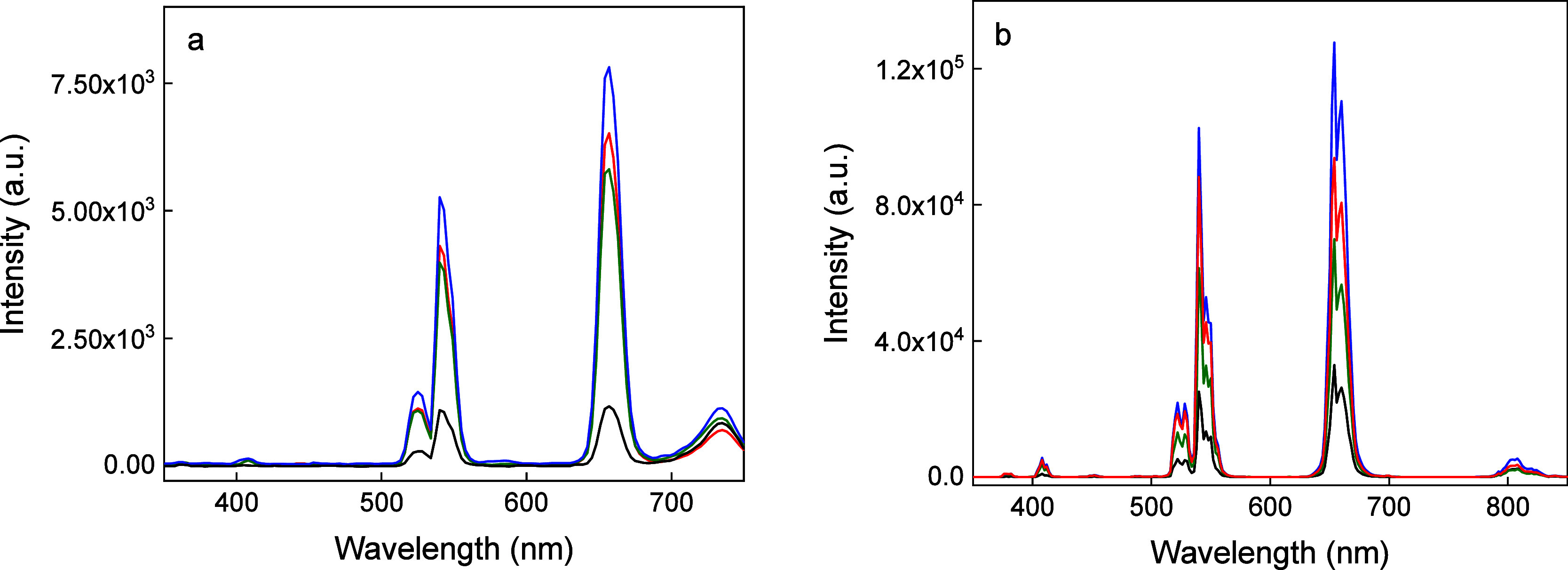
Upconversion photoluminescence spectra of C-UCNPs (black), CS-UCNPs (blue), CS-UCNP@PMVEMA (red), and CS-UCNP@PMVEMA-LGL nanoparticles (green) in water (1 mg/mL) excited at (a) 808 and (b) 980 nm with laser power densities of 3.5 and 2.11 W/cm^2^, respectively.

### Modification of CS-UCNPs with PMVEMA

To ensure good colloidal and chemical stability of hydrophobic CS-UCNPs in PBS and other physiological media and allow immobilization of biomolecules, the particles were thoroughly washed with hexane, ethanol, and water to remove excess oleic acid and octadec-1-ene from the surface and coated with a biocompatible PMVEMA polymer.^20,36^ The advantage of PMVEMA consists of the presence of a large number of carboxyl groups that can coordinate with the lanthanides on the particle surface, providing a strong attachment of the polymer. Modification of CS-UCNPs by PMVEMA was accompanied by a slight increase in their hydrodynamic diameter in water from 175 to 182 nm, but the particle size distribution remained narrow (*PD* = 0.17; Table 1). As expected, the size of CS-UCNP@PMVEMA in PBS increased to 230 nm (*PD* = 0.2) and then remained constant for 4 days without any particle sedimentation, demonstrating the superior colloidal stability of CS-UCNP@PMVEMA particles.^27^ At the same time, the ζ-potential became negative (−24 mV) due to the presence of ionized carboxyl groups (Table 1). The successful modification of the CS-UCNPs was documented by FTIR spectra, where characteristic peaks of PMVEMA including the absorption bands at 1709 and 1570 cm^–1^ were assigned to ν­(C=O) vibrations of COOH and COO^–^ groups (Figure 3a). The band at 1079 cm^–1^ was attributed to the symmetric stretching vibration ν_s_(–O–) of the OCH_3_ groups. The asymmetric ν_as_(CH_3_) and symmetric ν_s_(CH_2_) stretching vibrations were observed at 2926 and 2850 cm^–1^, respectively. According to TGA, the uncoated UCNPs contained 2.4 wt % residual oleic acid; the amount of PMVEMA on the surface of CS-UCNPs was 5.1 wt % (Figure 3b). At the same time, the modification of CS-UCNPs with PMVEMA slightly decreased their upconversion emission intensity under excitation at both 808 and 980 nm (Figure 2).

**Figure 3:**
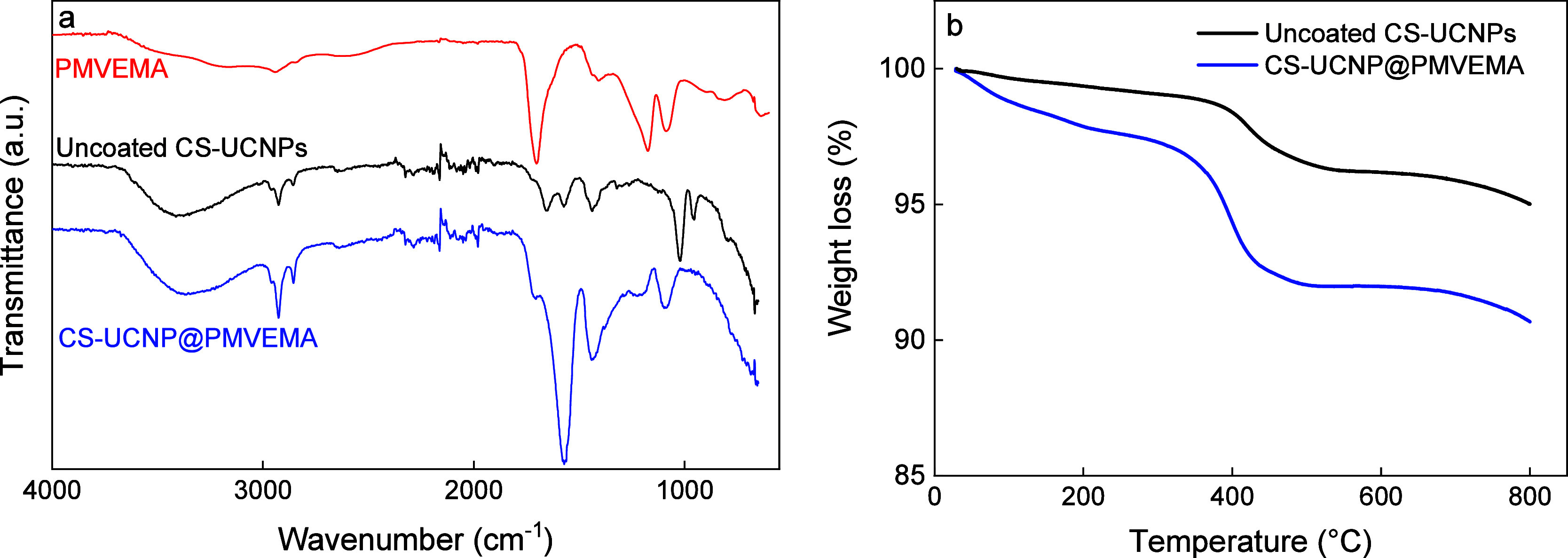
(a) ATR FTIR spectra and (b) TGA thermograms of uncoated and PMVEMA-coated CS-UCNPs.

### LGL-Conjugated PMVEMA-Coated CS-UCNPs (CS-UCNP@PMVEMA-LGL Nanoparticles)

The presence of abundant carboxyl groups in PMVEMA allowed not only coordination with the surface lanthanide ions of CS-UCNPs but also conjugation with the amino groups of target therapeutic agents such as peptides. In this report, LGL, a glucagon-like peptide-1 (GLP-1) analogue, was selected as a specific β-cell surface ligand. This cell-targeting peptide is highly expressed in pancreatic cells and is considered a promising agent for the delivery of theranostics to pancreatic β-cells and for diagnostics of their amount. LGL was conjugated to the CS-UCNP@PMVEMA nanoparticles by nucleophilic acyl substitution between the carboxyl group of the PMVEMA coating and the terminal amino group of LGL distant from the palmitoyl fatty acid chain with affinity for albumin, as described in the biological experiments below. It was necessary to control the pH of this reaction between 9.6 and 10.0 to deprotonate the amino groups of LGL (p*K*_a_ ∼ 9.5) and prevent LGL hydrolysis in an alkaline environment (pH > 10).^45^ After conjugation, the hydrodynamic particle diameter increased from 182 nm (*PD* = 0.17) to 258 nm (*PD* = 0.18); the ζ-potential decreased from −24 to −28 mV (Table 1). The observed shift of *D*_h_ and ζ-potential of CS-UCNP@PMVEMA-LGL particles was evidence of the successful conjugation of LGL with UCNP@PMVEMA nanoparticles. The *D*_h_ was also comparable to that of LGL-loaded chitosan, zein, and poly­(lactic-*co*-glycolic acid) nanoparticles used for oral drug delivery.^46–48^ The conjugation was further confirmed by a biuret colorimetric assay (reduction of Cu^2+^ to Cu^+^) used to detect peptide binding and UV–vis and fluorescence spectroscopy of CS-UCNP@PMVEMA-LGL particles (Figures 4 and S4). While CS-UCNP@PMVEMA nanoparticles were blue after reaction with biuret, LGL-containing particles turned purple, demonstrating peptide conjugation (Figure S4). As expected, the upconversion emission intensity of CS-UCNP@PMVEMA-LGL nanoparticles was weaker than that of neat and PMVEMA-coated CS-UCNPs due to the conjugation of LGL and the lower number of particles at the same concentration (Figure 2). Conjugation of CS-UCNP@PMVEMA particles with LGL resulted in an excitation peak at 297 nm (λ_em_ = 490 nm), whereas the spectrum of CS-UCNP@PMVEMA particles (without LGL) did not show the LGL peak (Figure 4a). The UV–vis spectrum of CS-UCNP@PMVEMA-LGL nanoparticles then showed a typical LGL shoulder peak in the 290–300 nm region (Figures 4b and S2). The chosen absorption wavelength of 290 nm provided a clear and reliable signal for the detection of LGL on CS-UCNP@PMVEMA particles. Note that the Saxenda formulation contained a phenol interfering at 280 nm, which increased the noise-to-signal ratio and prevented the observation of the absorption maximum of neat LGL.^49^ Its content was then 0.8 mg of LGL/mg of nanoparticles.

**Figure 4:**
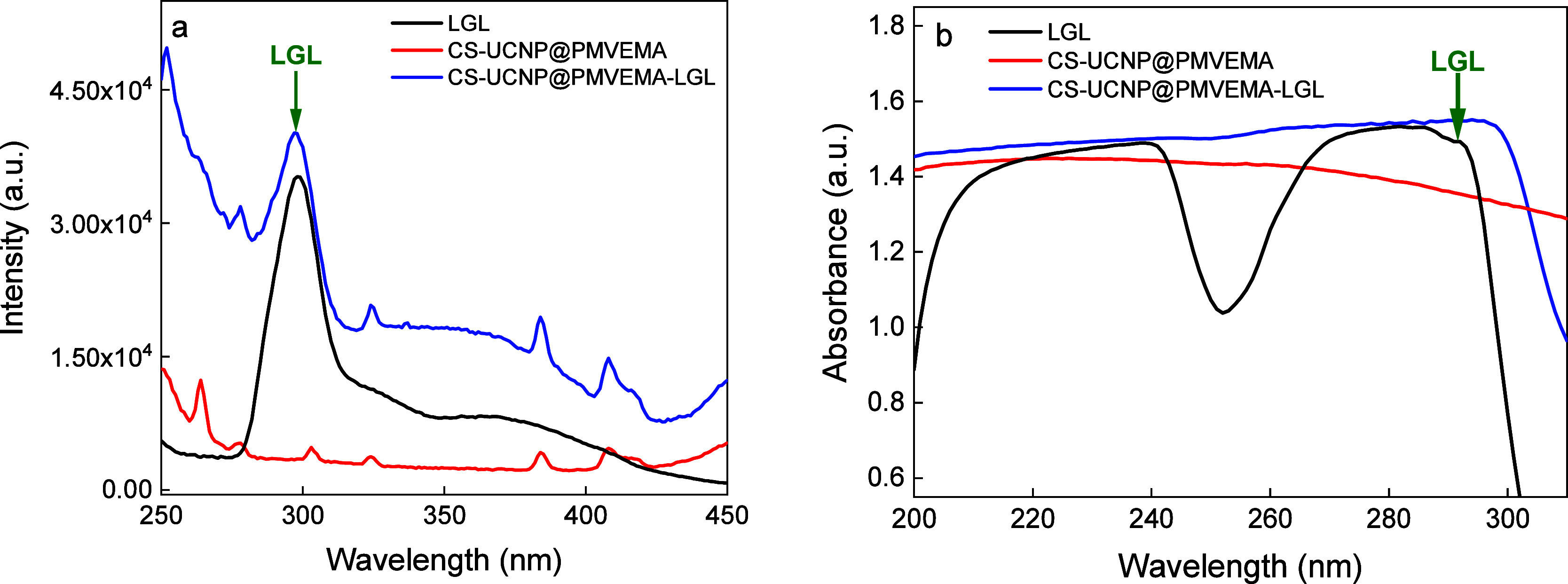
(a) Photoluminescence excitation (λ_em_ = 490 nm) and (b) UV–vis spectra of LGL (1 mg/mL) in borate buffer (pH = 10.6) and aqueous CS-UCNP@PMVEMA and CS-UCNP@PMVEMA-LGL dispersions (1 mg/mL). Green arrows indicate the LGL peaks.

### Cytotoxicity CS-UCNP@PMVEMA-LGL Nanoparticles

The toxicity evaluation of CS-UCNP@PMVEMA-LGL nanoparticles is crucial for their potential use in the treatment and imaging of the pancreas in living organisms. To verify the *in vitro* cytotoxicity of the particles, a trypan blue exclusion test was used after incubation with INS-1 cells for 2 and 24 h (Figure S5). Since cell viability was >95% after 24 h of incubation and the cells showed no damage, the nanoparticles could be considered nontoxic even at their high concentration (0.4 mg/mL); such concentration was much higher than those used *in vivo*. The superior biocompatibility of the CS-UCNP@PMVEMA-LGL nanoparticles was also evident when compared to the NaGdF_4_:Yb,Tb,Nd nanoparticles coated with poly­(4-styrenesulfonic acid-*co*-maleic anhydride) investigated for pancreatic islet imaging, which were cytotoxic already at a concentration of 0.1 mg/mL after 24 h incubation.^50^

### Amplification of Glucose-Stimulated Insulin Secretion in CS-UCNP@PMVEMA-LGL-Labeled Perifused Pancreatic Islets

The primary function of pancreatic β-cells is insulin production and secretion, which can best be illustrated as glucose-stimulated insulin secretion (GSIS). Experimental GSIS is preferably monitored by perifusion of pancreatic islets, which allows quantification of the rate of insulin release.^51^ To quantify the rate of insulin release during GSIS, perifusion was first examined with a low glucose concentration (2.5 mM) to observe basal (near zero) insulin secretion and then with a high glucose concentration (25 mM), which is commonly used to mimic postprandial state (Figure 5). Since GLP-1 or its analogues are known to increase GSIS, we tested whether LGL as an analogue of the GLP-1 peptide retains its function when conjugated to CS-UCNP@PMVEMA nanoparticles added to the perifusion medium. The results showed that CS-UCNP@PMVEMA-LGL nanoparticles amplified the GSIS, demonstrating that the LGL-conjugated nanoparticles remained functional and interacted with the GLP-1 receptor (GLP1R; Figure 5). CS-UCNP@PMVEMA particles without conjugated LGL used as a control did not increase GSIS. Positive control then consisted of treating glucose-stimulated islets with a depolarizing concentration of KCl (30 mM), which resulted in a transient release of insulin, confirming that the particles did not affect the cell function after contact with GLP1R.

**Figure 5:**
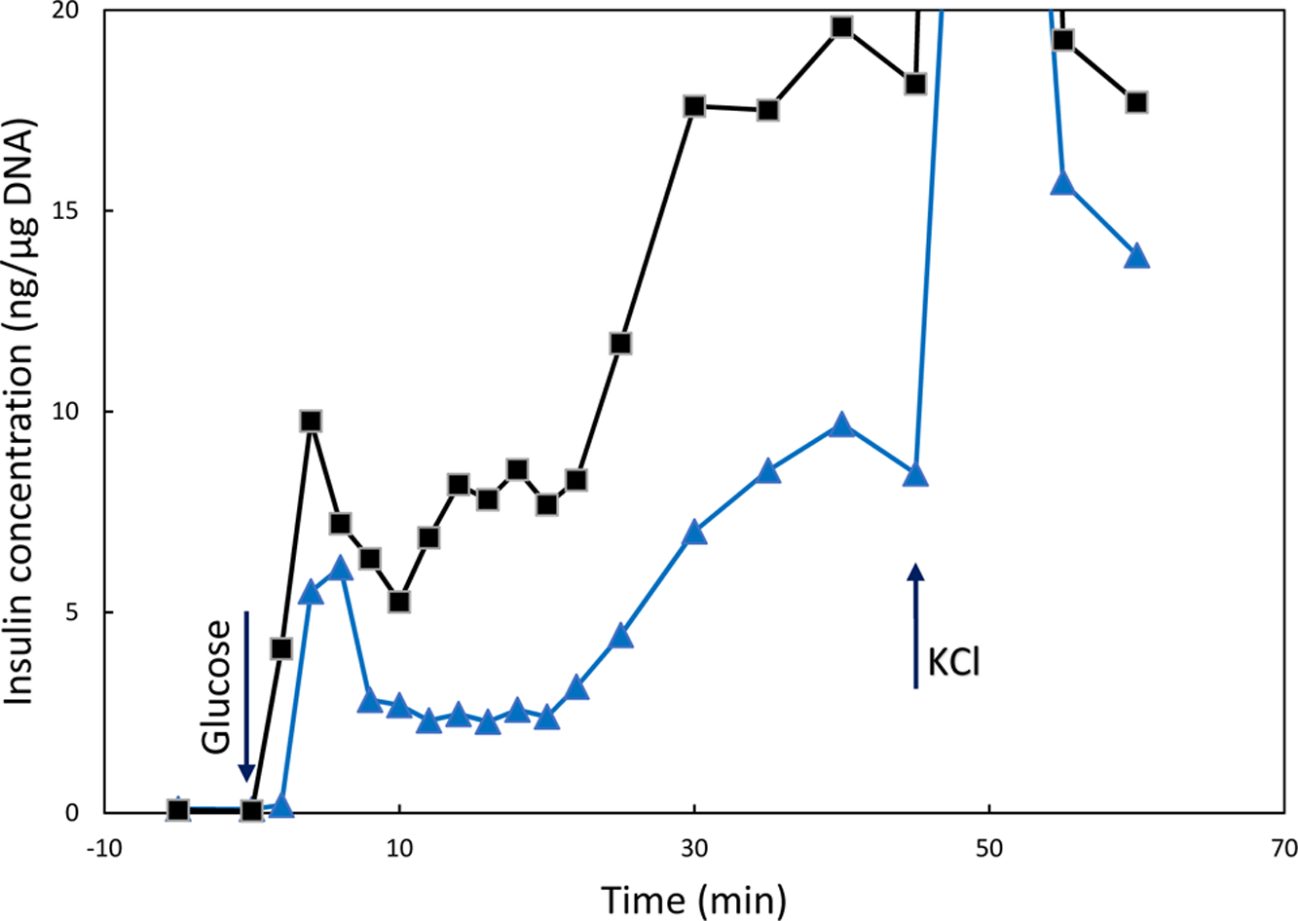
Insulin secretion from isolated pancreatic islets incubated with (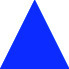) CS-UCNP@PMVEMA and (■) CS-UCNP@PMVEMA-LGL nanoparticles after stimulation with glucose added at the beginning of the experiment (arrow). KCl was added to both runs after 45 min to show maximum insulin releasing capacity.

### Binding of CS-UCNP@PMVEMA-LGL Nanoparticles to GLP1R of β-Cells of Pancreas

As any cell tends to engulf foreign species by endocytosis, it can be assumed that also LGL-conjugated CS-UCNP@PMVEMA nanoparticles as well as LGL-free particles will be captured on the GLP1R of model cells but also the pancreatic islet β-cell surface. The latter should bind LGL-conjugated nanoparticles and internalize them after binding to GLP1R.^52^ As a result, their uptake into or “labeling” of β-cells should be higher than for LGL-free nanoparticles. Within a sufficiently long time, exocytosis of any nanoparticles can also occur; therefore, the dynamic equilibrium between endocytosis *vs*. exocytosis, or particle binding *vs*. release from the receptor must be considered. The internalization of CS-UCNP@PMVEMA-LGL nanoparticles was tested after their incubation with INS-1E cells (Figure 6a). The particles bound to cell membranes were also engulfed, confirming the targeting of LGL-conjugated nanoparticles to GLP1R in model pancreatic β-cells.

**Figure 6:**
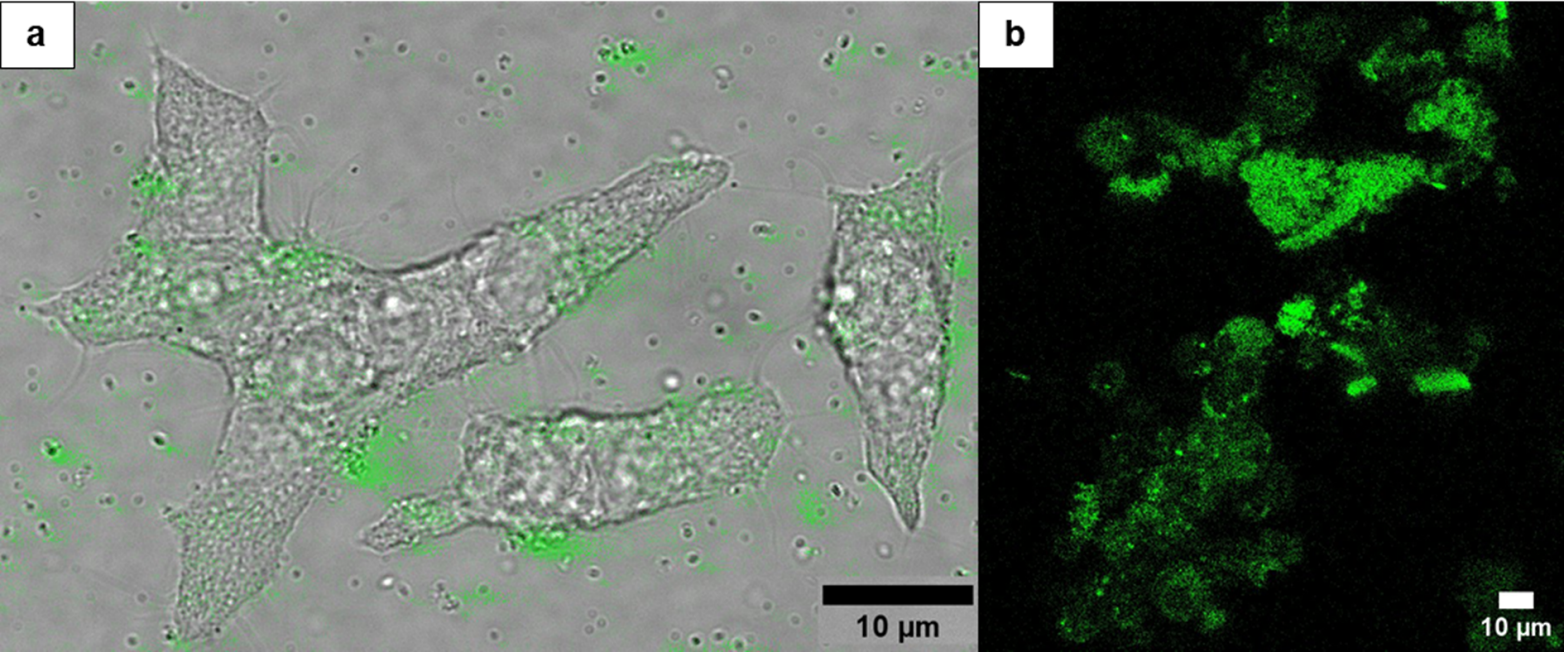
Confocal micrographs of (a) INS-1E cells and (b) pancreatic islets incubated with CS-UCNP@PMVEMA-LGL nanoparticles for 24 h after 980 nm excitation.

To see the interaction of β-cells with CS-UCNP@PMVEMA-LGL nanoparticles, they were incubated with pancreatic (Langerhans) islets for 15 min and 24 h and stained for TEM imaging. The β-cells of pancreatic islets were well recognizable on the micrographs due to the presence of many insulin granules, and the distribution of particles was also discernible at both time points (Figure 7). While 15 min after incubation, the CS-UCNP@PMVEMA-LGL nanoparticles were visible in the proximity of the cell surface and were not endocytosed (Figure 7a), after 24 h, the nanoparticles were localized predominantly inside endosomes (Figure 7b,c), unlike nanoparticles without LGL (Figure 7d).

**Figure 7:**
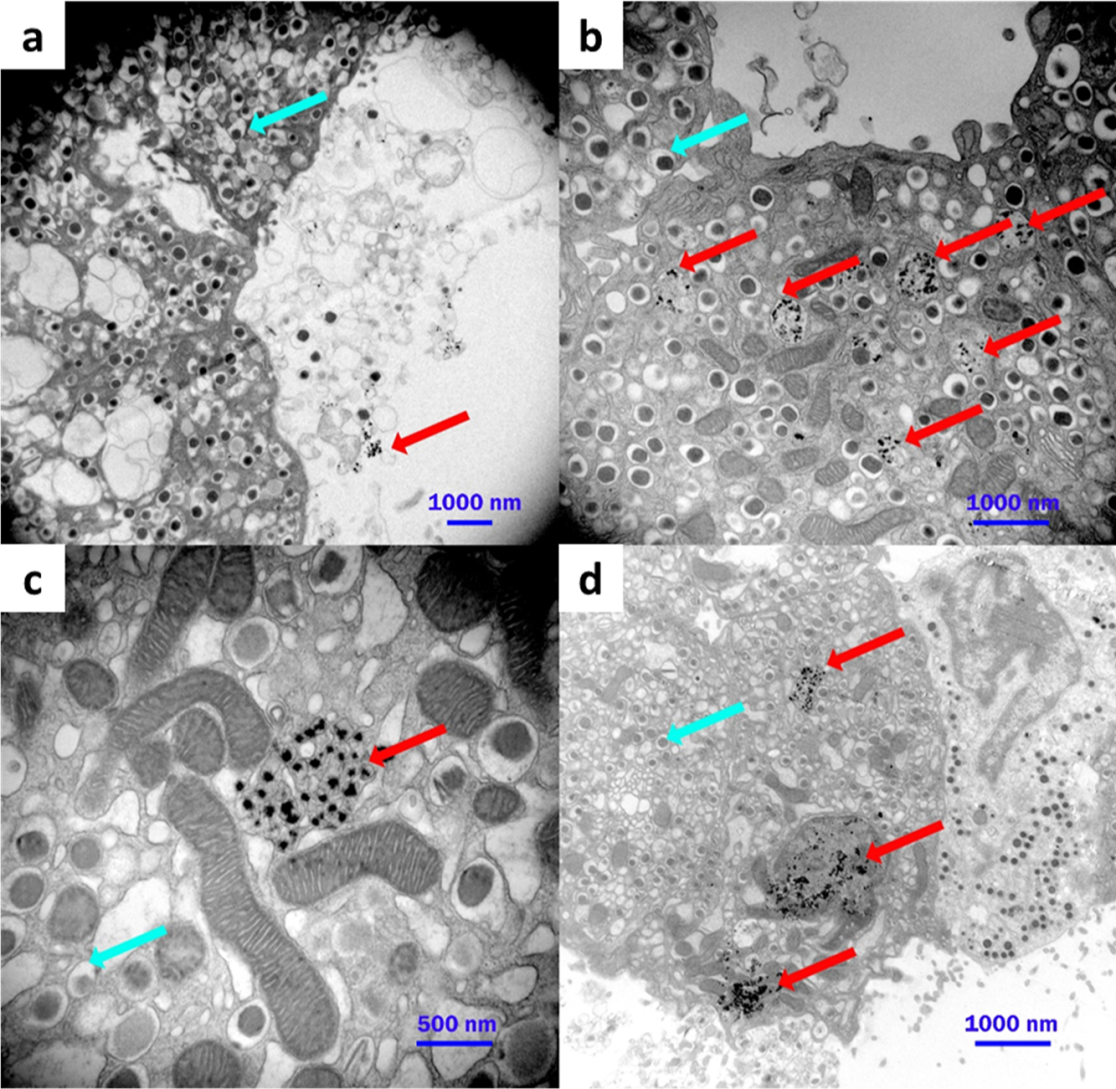
TEM micrographs of (a–c) CS-UCNP@PMVEMA-LGL-labeled β-cells and (d) cells of a single Langerhans islet incubated with nanoparticles without LGL (a) 15 min and (b–d) 24 h after incubation. Red arrows show the nanoparticles and cyan arrows show insulin granule vesicles.

Upconversion luminescence of pancreatic islets incubated with CS-UCNP@PMVEMA-LGL nanoparticles was verified by upright confocal microscopy at 808 and 980 nm excitation (Figures 6b and S6). The advantage of 980 nm excitation is the low autofluorescence of cells, while 808 nm excitation also allows deeper penetration of light into tissues. Under both excitations, upconversion light was emitted at wavelengths of 533 and 647 nm (Figure S6), which was also later observed in the extracted pancreas of mice after intramuscular administration of CS-UCNP@PMVEMA-LGL nanoparticles (Figure 8a–i). No other biological luminescence exhibits such properties, so the approach we use examines nanoparticles with high fidelity. Histological sections of the pancreas showed islets of Langerhans with bound nanoparticles (Figure 8c,d). Monitoring of the pancreas using upright confocal microscopy reflected the pharmacokinetics of the nanoparticles in the body (Figure 8a–i). Fifteen minutes after intramuscular administration, small amounts of nanoparticles accumulated in the pancreas (Figure 8e,f) but were also detected in other organs such as the liver and kidney (Figure 8j,k). However, 24 h after administration, CS-UCNP@PMVEMA-LGL nanoparticles accumulated exclusively in the pancreas (Figure 8d,g,h), while the corresponding images of the liver, kidney, and other organs such as the lung were completely dark (images not shown). This suggests that the particles specifically accumulated in the pancreas, confirming the binding between LGL and GLP1R of β-cells.

**Figure 8:**
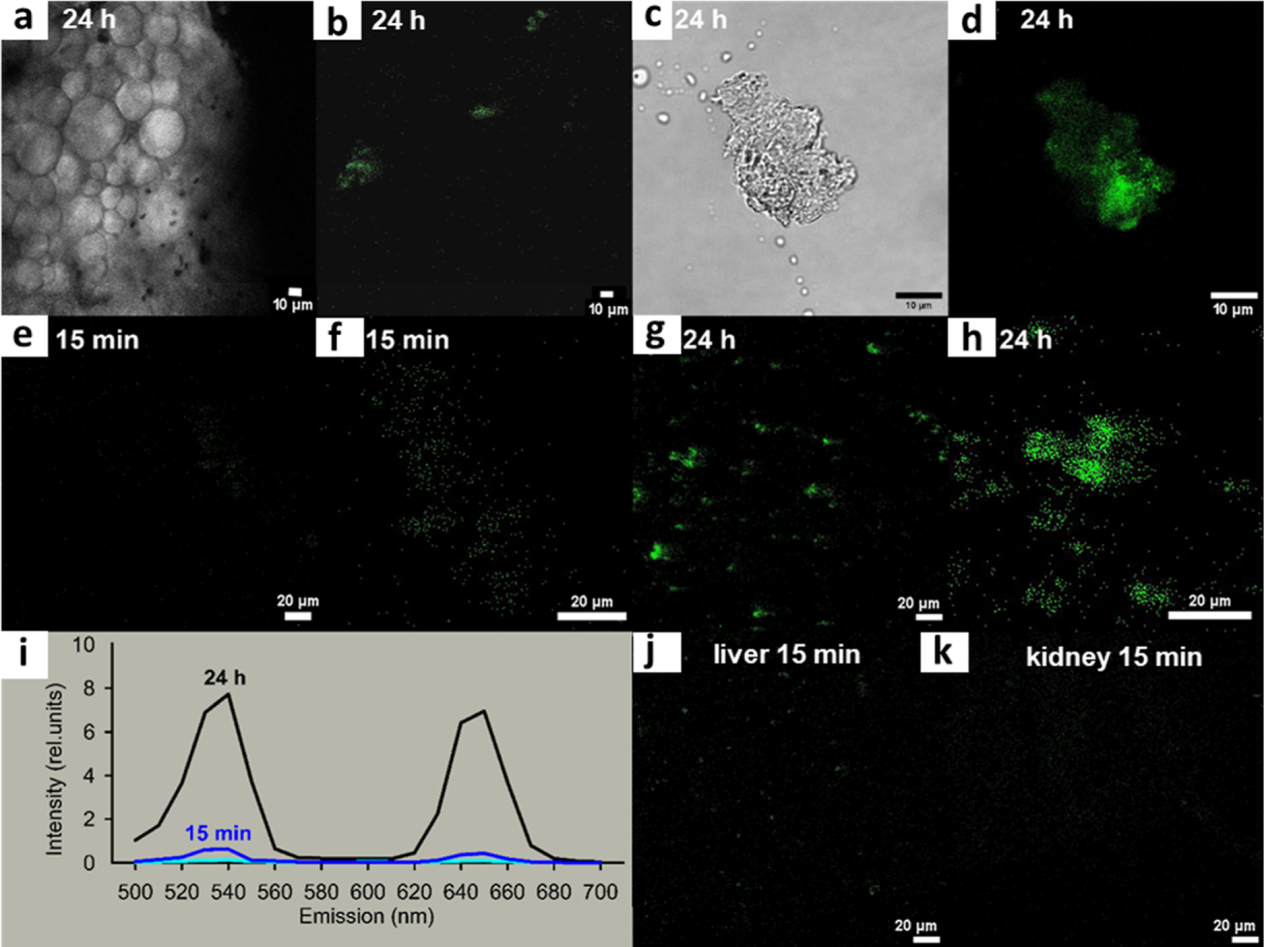
(a–h) Confocal micrographs of mouse pancreas taken by an upright objective in the indicated time intervals after intramuscular injection of CS-UCNP@PMVEMA-LGL nanoparticles; (c,d) pancreas was excised, (a,c) phase contrast at 405 nm illumination, (b,d, e–h) pancreas at 980 excitation, (e,g) imaged in a widefield and (f, h) focused at islets with nanoparticles (green). (i) Upconversion emission spectra in the pancreas 15 min (blue) and 24 h (black) after nanoparticle administration and for the background (light blue). Confocal micrographs of (j) liver and (k) kidney taken 15 min after nanoparticle administration. Images of liver, kidney, and other organs (lung) were completely dark after administration.

In contrast, when the CS-UCNP@PMVEMA-LGL nanoparticles were administered intravenously, they were not found in the pancreas. This may be explained by the fact that they were captured by macrophages.^53^ In the case of intramuscular administration, the slow release of particles from the injection sites and the contribution of the lymphatic system should be considered. This agrees with the literature where intraperitoneal delivery of polymeric nanoparticles resulted in almost 15-times higher accumulation in the pancreatic tumor than intravenous injection.^54,55^ In addition, the binding of LGL to albumin enabled prolonged circulation of CS-UCNP@PMVEMA-LGL nanoparticles in the bloodstream.^56^ Last but not least, the affinity of albumin for LGL-conjugated nanoparticles indicated that LGL did not lose its activity.

The above results were also corroborated by ICP–MS elemental analysis of the excised pancreas after both intramuscular and intravenous administration of particles (Table S2). Quantification of rare earth elements in pancreas 24 h after injection showed that intramuscularly administered CS-UCNP@PMVEMA-LGL nanoparticles accumulated in the pancreas significantly more (∼89 mg Y^3+^/kg and ∼66 mg Yb^3+^/kg) compared to control CS-UCNP@PMVEMA nanoparticles (15 μg Y^3+^/kg and 8 μg Yb^3+^/kg) or intravenously administered CS-UCNP@PMVEMA-LGL nanoparticles (124 μg Y^3+^/kg and 91 μg Yb^3+^/kg). Although no undesirable side effects were observed after either intravenous or intramuscular injection of particles, intramuscular injection was preferred over intravascular administration considering the targeting of particles to the pancreas.

### Distribution of CS-UCNP@PMVEMA-LGL-Flamma Nanoparticles in Mice

In order to visualize the distribution of nanoparticle in the mouse body over time using optical imaging, the Flamma NIR fluorescent probe was bound to the CS-UCNP@PMVEMA-LGL nanoparticles *via* the reaction of its hydrazide groups with the carboxyl groups of PMVEMA. The advantage of Flamma is that it has a wide spectral range from the UV to the NIR region, high absorption, and quantum yield and is photostable. Even after Flamma binding, its fluorescence was not compromised (Figure S7), so it is easy to image also low-abundant biomolecules. After conjugation of Flamma, the *D*_n_ and *D*_h_ values of the particles were similar to those before binding (Table 1), indicating that the attachment of the dye did not affect the particle morphology. The ζ-potential of the particles decreased from −28 to −36 mV due to the anionic nature of the sulfo groups of the dye (Table 1). The observed ζ-potential shift of CS-UCNP@PMVEMA-LGL-Flamma nanoparticles thus demonstrated the conjugation of Flamma to the surface of CS-UCNP@PMVEMA-LGL nanoparticles. The presence of Flamma dye on the particles was also demonstrated by photoluminescence spectra (Figure S7). While the excitation and emission maxima of the free Flamma were observed at 749 and 774 nm, respectively, the spectra of CS-UCNP@PMVEMA-LGL-Flamma nanoparticles showed a shift of the excitation (by 9 nm) and emission peak (by 6 nm), which is further evidence of the successful conjugation of Flamma to the particles. Conjugation of Flamma to CS-UCNP@PMVEMA-LGL nanoparticles did not affect their upconversion luminescence at both 808 and 980 excitations (Figure S8).

For biodistribution experiments, two black and two nu/nu mice were selected. The use of these different mouse models was motivated by two main objectives: (*i*) to compare *in vivo* signal absorption between black-furred mice, in which melanin in the fur may absorb a significant portion of the signal, and nude (hairless) mice, which minimize external interference to absorption, and (*ii*) to investigate the effect of different immune system profiles on the biodistribution and accumulation of injected nanoparticles. Nu/nu mice, which are characterized by specific immunodeficiency due to the absence of functional T-cells and impaired B-cell activity, were included in the study to evaluate whether their immunocompromised state affects biodistribution patterns compared to immunocompetent black mice. The aim of this comparison was to determine potential differences in nanoparticle uptake and accumulation in systemic and pulmonary tissues. In both cases, however, the selection of mice had no significant effect on both signal absorption and nanoparticle biodistribution.

The CS-UCNP@PMVEMA-LGL-Flamma nanoparticles were administered to mice by intramuscular and/or intravenous injection and their *in vivo* biodistribution was monitored at 10, 60, and 180 min and 24 h postinjection (Figure 9). Fluorescence imaging using the Light Imager revealed a detectable signal in the pancreatic region 10 min to 1 h after injection. However, this signal could not be clearly anatomically localized, and although some slight variations were observed, they were not significant. Based on these initial findings, a longer postinjection interval of 3 h was chosen for further investigation. At this time interval, imaging showed more pronounced changes in the distribution and intensity of the fluorescent signal with clearer anatomical localization to the pancreas (Figure 9a,c). Subsequently, the mice were sacrificed after 3 h to confirm the presence and accumulation of nanoparticles in the pancreatic tissue (Figure 9b,d). To assess potential long-term retention or clearance of the particles, an additional imaging experiment was performed 24 h after injection (Figure 9e,g). This extended observation period provided a more comprehensive view of the nanoparticle uptake and biodistribution. Organ dissection was also performed after 24 h to allow direct comparison of fluorescence signals in different organs (Figure 9f,h). The optical imaging at the time points of 3 and 24 h confirmed a strong correlation between *in vivo* signal retention and organ-specific nanoparticle distribution. Based on these findings, only the 3 h (which showed the most intense early signal) and 24 h time points were presented (Figure 9). It should also be noted that no adverse effects were observed in mice when CS-UCNP@PMVEMA-LGL-Flamma nanoparticles were administered intravenously or intramuscularly.

**Figure 9:**
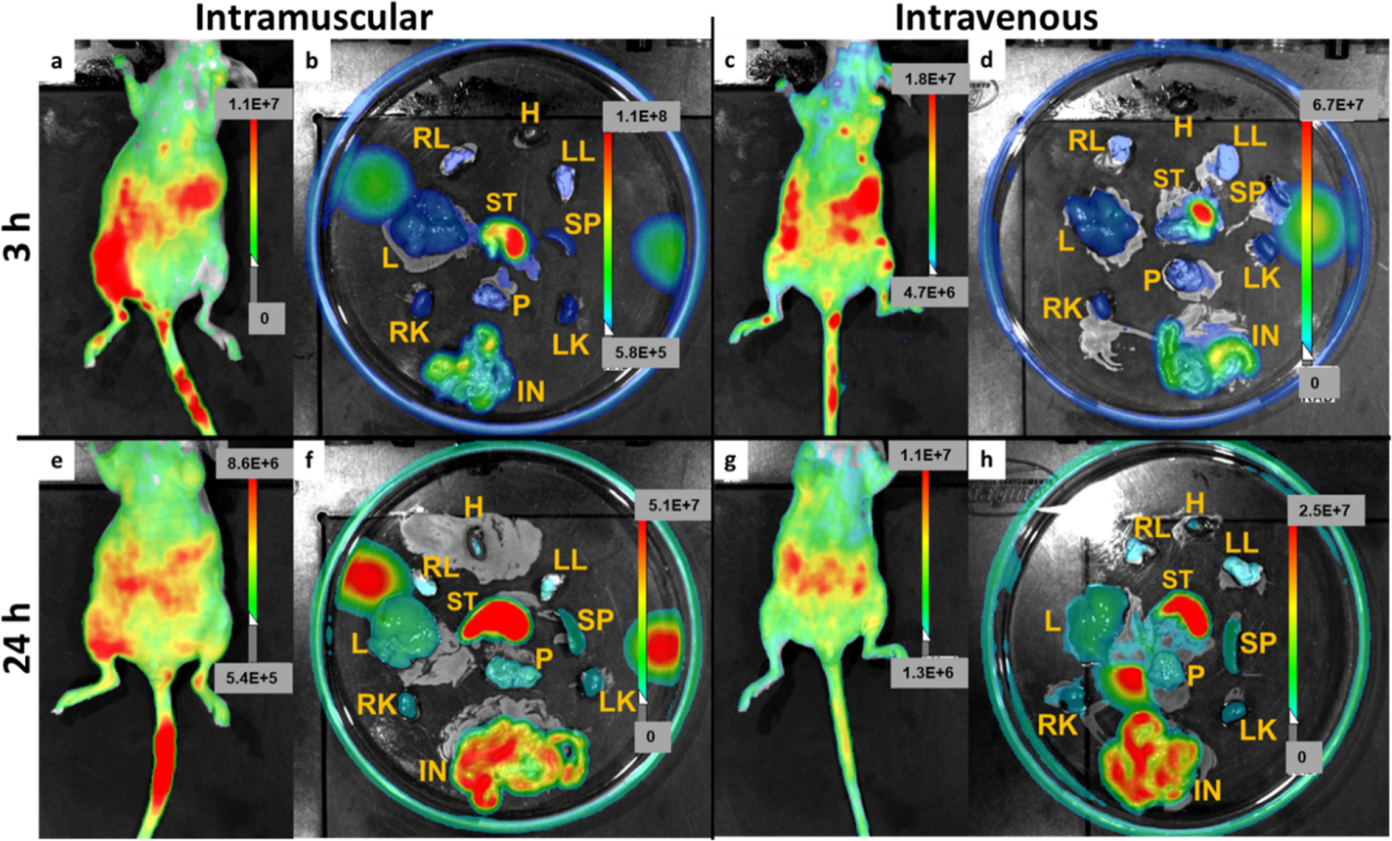
*In vivo* optical imaging of CS-UCNP@PMVEMA-LGL-Flamma nanoparticle biodistribution after (a–d) 3 h using black mice and (e–h) 24 h using nu/nu mice; (a,b,e,f) intramuscular and (c,d,g,h) intravenous administration. H—heart, RL—right lung, LL—left lung, SP—spleen, L—liver, ST—stomach, P—pancreas, LK—left kidney, RK—right kidney and IN—intestine. Luminescence units are in radians (photons/s/cm^2^).

Intramuscular injection of CS-UCNP@PMVEMA-LGL-Flamma nanoparticles revealed a time-dependent biodistribution pattern. At 10 min postinjection, a weak nonspecific fluorescent signal was observed with a low probability of accumulation of particles in pancreas. The signal was predominantly located in the gastrointestinal organs, including the stomach and intestines as well as the bladder and possibly the liver. After 1 h, the signal became stronger and more anatomically defined, suggesting an increased likelihood of particle penetration in the pancreas. The specificity of the signal was also improved, which was clearly detected in the liver, bladder, and muscle tissue, including peripheral areas. Within 3 h, *in vivo* imaging confirmed specific accumulation of nanoparticles in the lungs (thoracic region), pancreas, liver, intestines, stomach, bladder, kidneys, and large muscle tissues (Figure 9a,b). This distribution pattern indicated preferential uptake of these organs. After 24 h, *in vivo* analyses demonstrated continued retention of nanoparticles in the lungs, heart, stomach, pancreas, liver, kidneys, bladder, and large muscle groups (Figure 9e,f). The consistent detection of signals in these specific organs at a later time point (24 h) highlighted preferential accumulation of nanoparticles in these regions. In the case of intravenous injection of CS-UCNP@PMVEMA-LGL-Flamma nanoparticles, the *in vivo* signals from animal organs 3 h (Figure 9c,d) and 24 h after administration (Figure 9g,h) were similar to those observed after intramuscular application.

A direct comparison of intramuscular and intravenous administration of nanoparticles to the mouse pancreas revealed a noteworthy finding. At 3 h postinjection, the signal intensity in the pancreas was the same for both routes of administration, with a measured value of 2.6 × 10^7^ photons/s/cm^2^ (Figure S9a,b). However, after 24 h, intramuscular injection resulted in a significantly higher signal intensity in the pancreas (6.3 × 10^7^ photons/s/cm^2^) compared to intravenous injection (3.4 × 10^7^ photons/s/cm^2^; Figure S9c,d). This increased accumulation after intramuscular administration was likely due to the proximity of the injection site (outer thigh) to the pancreas and the slow and sustained release of nanoparticles from muscle tissue.^57,58^ In contrast, intravascular administration was associated with a short *in vivo* half-life of LGL and preferential binding to proteins and lipoproteins.^53,55^ Remarkably, parenteral administration, such as subcutaneous, intramuscular, or intravenous injections, is widely used for the administration of peptide-based drugs because it avoids the biological barriers of oral and pulmonary administration. Previous studies of the *in vivo* distribution of amino-modified silica nanoparticles loaded with LGL and fibroblast growth factor-21 showed their passive accumulation mainly in the liver 24 h after intravenous postinjection.^12^ In contrast, negligible fluorescence signals were observed in other organs such as spleen, lung, kidney, heart, stomach, intestine, and colon due to their relatively low accumulation and limited depth of penetration of fluorescence imaging. Other strategies investigated for targeting β-cells *in vivo* *via* parenteral injection have used chitosan or iron oxide nanoparticles functionalized with the GLP-1 mimetic exendin-4 and decorated with the organic dye.^59,60^ These nanoparticles have demonstrated the ability to target the pancreas *via* β-cell GLP-1 receptors, reducing hepatic and renal accumulation and allowing multimodal detection. The advantage of our *in vivo* delivery of CS-UCNP@PMVEMA-LGL nanoparticles is that it demonstrated great potential for monitoring drug pharmacokinetics, diabetes mellitus treatment, and β-cell imaging.

Comparing the biodistribution of CS-UCNP@PMVEMA-LGL nanoparticles with some literature data, similar behavior has been reported for clinically approved superparamagnetic iron oxide (SPIO) nanoparticles, such as Resovist and Endorem. These nanoparticles were primarily processed by the mononuclear phagocyte system, leading to their accumulation in organs such as the liver, spleen, and pancreas.^61^ The particles were internalized by macrophages and metabolized into iron ions, which were integrated into physiological pathways, including hemoglobin and ferritin synthesis.^62^ Alternatively, excretion of SPIO particles occurred *via* the biliary tract into the feces and to a lesser extent *via* renal clearance.^63^ Resovist and Endorem have been shown to enhance contrast of transplanted pancreatic islets in magnetic resonance images.^62,64,65^ The mechanisms of cellular uptake and retention of SPIO particles have also been investigated with respect to their potential for targeted imaging and drug delivery.^66^ Thus, the biocompatibility and utility of magnetic nanoparticles as imaging agents have provided the basis for the development of novel functionalized particles for theranostics such as CS-UCNP@PMVEMA-LGL.

## Conclusions

This report proposes a new upconversion system with a hexagonal phase based on monodisperse core UCNPs doped with Yb^3+^, Er^3+^, and Fe^2+^. The presence of Fe^2+^ ions and the introduction of a NaYF_4_:Nd shell on the core particles induced dominant upconversion emission in the red region and increased luminescence at excitation wavelengths of 808 and 980 nm. Another novelty, which according to our information has not yet been published, is the covalent binding of LGL to PMVEMA-coated CS-UCNPs with the aim of using them in diabetes theranostics. Coating of particles by PMVEMA ensured their dispersibility in water and PBS, while conjugation of LGL, a glucagon-like peptide-1 analogue, by EDC/NHS coupling chemistry allowed activation of GLP-1 receptors of the pancreatic β-cells, increasing insulin secretion. The nanoparticles demonstrated nontoxicity, with cell viability exceeding 95% even at high concentrations, as confirmed by trypan blue exclusion assay. Optionally, the Flamma dye was attached to the particles to facilitate their localization in the mouse body using an optical microscope. An interesting finding was that the biodistribution of particles was affected by the route of administration. This was the first time that *in vivo* intravenous and intramuscular administration of LGL transported on a carrier into the pancreas was compared. Based on the *in vivo* results, the luminescence signal of CS-UCNP@PMVEMA-LGL-Flamma nanoparticles in the pancreas was clearly stronger 24 h after intramuscular administration (6.34 × 10^7^ photons/s/cm^2^) than after intravenous injection (only 3.42 × 10^7^ photons/s/cm^2^). Monitoring the biodistribution and accumulation behavior of CS-UCNP@PMVEMA-LGL nanoparticles is thus a good indicator for considering their utilization in pharmacokinetics and as a drug carrier in clinical trials. However, further studies are needed to thoroughly explore these possibilities and to extend the characterization and evaluation of the particles to other animal models.

## Supplementary Material



## Notes

Concentrations of rare earth ions in C- and CS-UCNPs and pancreas by AAS and ICP–MS analysis; scheme of Flamma 749 hydrazide; UV–vis absorption spectra and calibration curve of desalted LGL; TEM characterization; biuret test; viability of cells; upconversion emission spectra of pancreatic islets; photoluminescence excitation and emission spectra; and fluorescence imaging of particles in pancreas (PDF)
