# Supplementary material for: Liraglutide-Conjugated Poly(methyl vinyl ether-alt-maleic acid)-Coated Core–Shell Upconversion Nanoparticles for Theranostics of Diabetes
Source: ACS Appl Mater Interfaces. 2025 Jul 16;17(30):42863–76. doi: 10.1021/acsami.5c11275 (PMC12314866; doi:10.1021/acsami.5c11275)
Supplement: Supplementary file 1 [file am5c11275_si_001.pdf]

Supporting Information

Liraglutide-conjugated poly(methyl vinyl ether-*alt*-maleic acid)-coated core-shell upconversion nanoparticles for theranostics of diabetes

Oleksandr Shapoval[1,], Hana Engstová[2], Miroslav Šlouf[1], Olga Kočková[1], Andrea Dlasková[2], Martin Jabůrek[2], Aminadav Halili[3], Alexandra Mozheitová[2], Daniel Jirák[3,4,5], Petr Ježek[2], Daniel Horák[1,*]*

[1] Institute of Macromolecular Chemistry of the Czech Academy of Sciences, Heyrovského nám. 2, 162 00 Prague 6, Czech Republic
[2] Institute of Physiology of the Czech Academy of Sciences, Vídeňská 1083, 142 20 Prague 4, Czech Republic
[3] Institute for Clinical and Experimental Medicine, Vídeňská 1958/9, 140 21 Prague 4, Czech Republic
[4] Institute of Biophysics and Informatics, First Faculty of Medicine, Charles University, 12108 Prague 1, Czech Republic
[5] Faculty of Health Studies, Technical University of Liberec, 461 17 Liberec, Czech Republic

[*]Corresponding authors. horak@imc.cas.cz, shapoval@imc.cas.cz

Table S1. Concentrations of metal ions in C- and CS-UCNPs determined by AAS and ICP-MS.

Metal ion concentration	C-UCNPs (mol.%)	CS-UCNPs (mol.%)
Y^{3+}	62	70
Yb^{3+}	20	14
Er^{3+}	15	10
Nd^{3+}	-	6
Fe^{2+}	3	*

* Below the detection limit of measurement according to AAS.

Table S2. Concentrations of rare earth ions in pancreas 24 h after intramuscular and intravenous administration of particles determined by ICP-MS analysis.

Particles	Administration	Y^{3+} (µg/kg)	Yb^{3+} (µg/kg)
CS-UCNP@PMVEMA	Intramuscular	15	8
CS-UCNP@PMVEMA-LGL	Intramuscular	89146	65974
	Intravenous	124	91

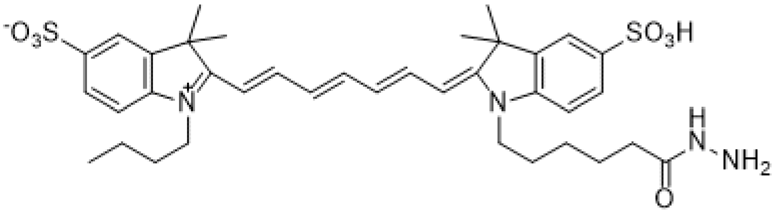

Figure S1. Flamma® 749 hydrazide.

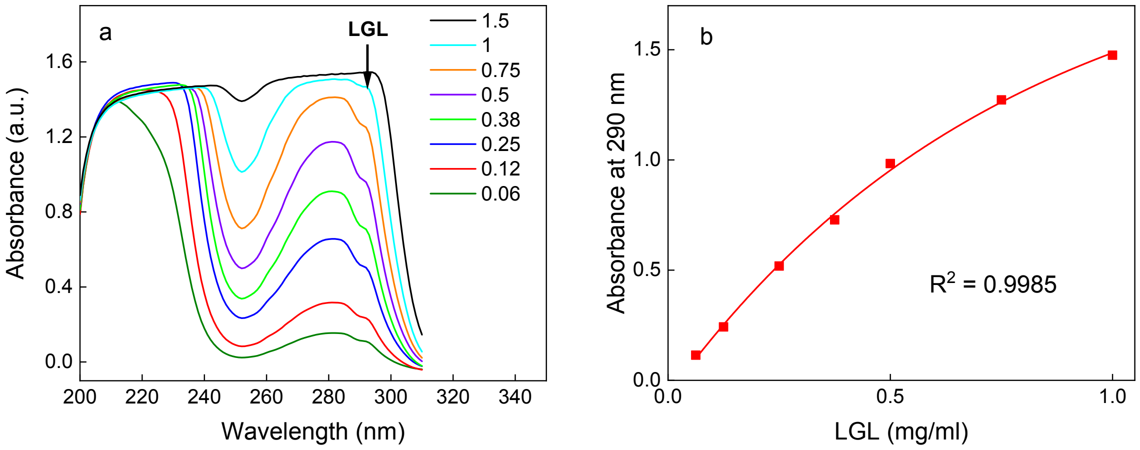

Figure S2. (a) UV-Vis absorption spectra and (b) calibration curve of desalted LGL (0.06-1.5 mg/ml) in borate buffer (pH = 10.6). Black arrow shows the LGL peak at 290 nm.

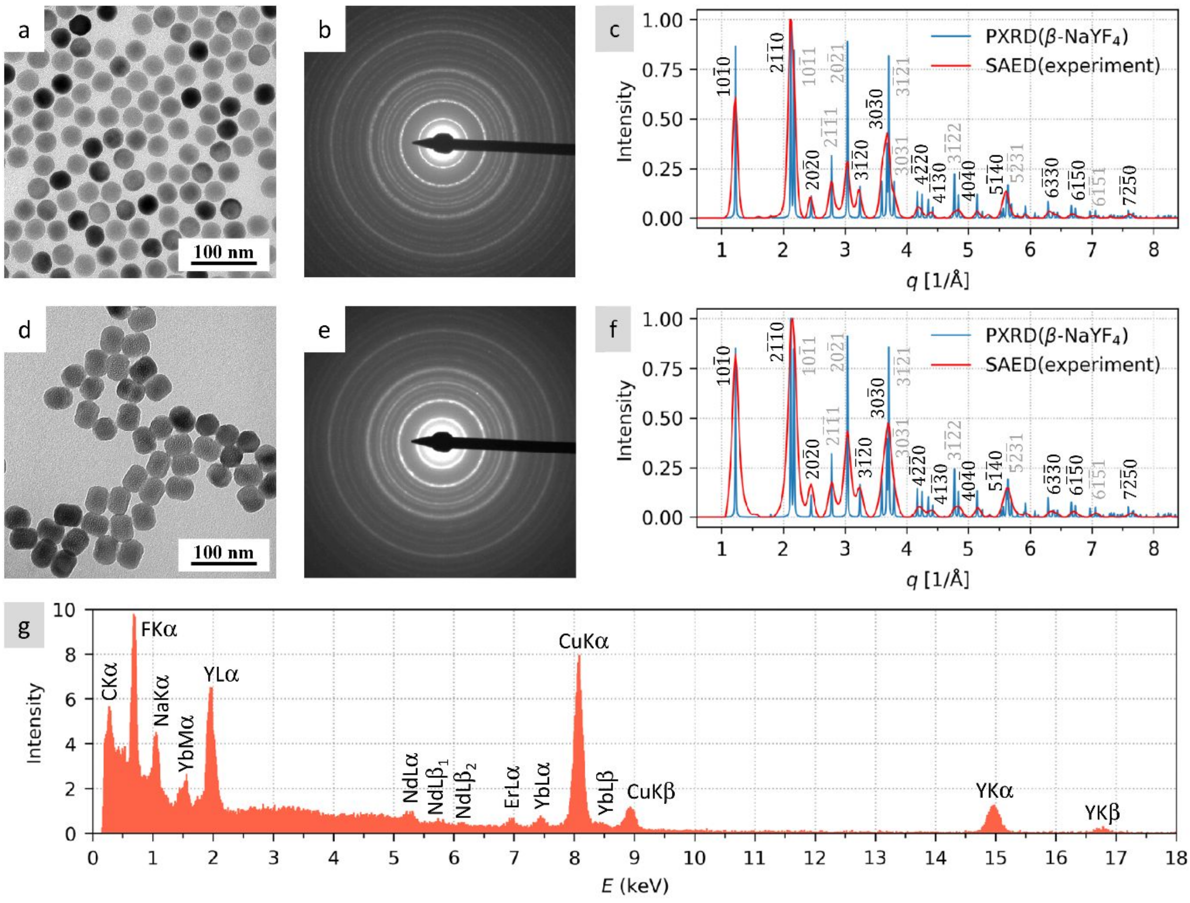

Figure S3. TEM characterization of (a-c) C-UCNPs and (d-g) CS-UCNPs. (a, d) TEM/BF micrographs of the nanoparticles, (b, e) experimental TEM/SAED diffraction patterns, (c, f) comparison of the radially averaged TEM/SAED diffraction patterns (red lines) with

theoretically calculated PXRD patterns of hexagonal β-$NaYF_4$ crystals (blue lines), and (g) TEM/EDX spectrum of the CS-UCNPs.

The comparison of the radially-averaged TEM/SAED diffractograms with the theoretically calculated PXRD patterns proved that both C-UNCPs and CS-UNCPs exhibited the crystal structure of hexagonal phase of sodium yttrium fluoride (β-$NaYF_4$; Figure S3 c and f). The positions of the SAED and PXRD diffraction peaks were in perfect agreement, while the intensities of the diffractions showed certain differences. Detailed comparison of the SAED and PXRD diffraction patterns revealed that the differences resulted from a preferred orientation (also referred to as texture) of both C-UNCPs and CS-UNCPs. It should be noted that the most intensive SAED diffractions (Figures S3 c and f) had Bravais indices (*hkil*) ending with zero, i.e. the most intensive experimental diffractions took the form *hki0* (marked with black font in Figures S3 c and f). This indicated that a high number of nanocrystals on the carbon film were preferentially oriented, exhibiting zone axis with Weber indices *uvtw* = 0001 and thus laying on 0001 crystallographic plane that was perpendicular to *c* axis (the more general Miller indices of the zone axis (*uwv*) and the plane (*hkl*) would be equal to 001). An analogous preferred orientation was observed in our previous study,[1] giving a more detailed justification based on Weiss zone law.[2]

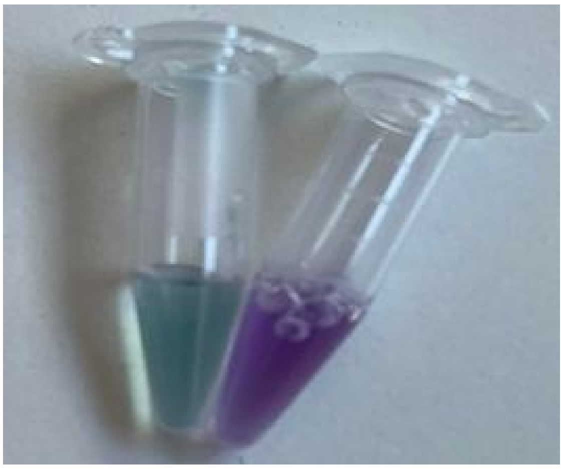

Figure S4. The biuret test of CS-UCNP@PMVEMA (left) and CS-UCNP@PMVEMA-LGL nanoparticles (right).

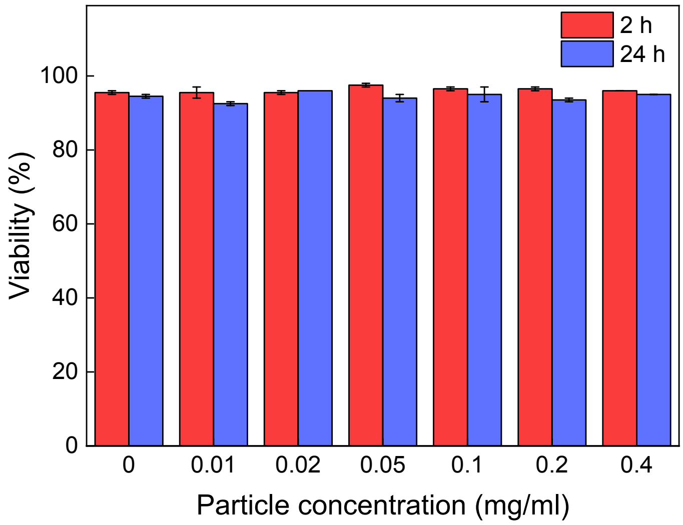

Figure S5. Concentration-dependent viability of INS-1E cells incubated with CS-UCNP@PMVEMA-LGL nanoparticles for 2 h (red) and 24 h (blue).

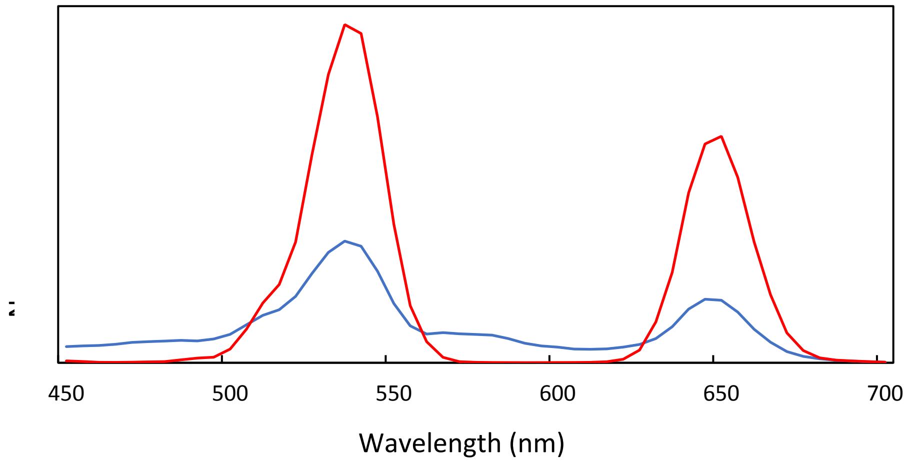

Figure S6. Upconversion emission spectra of pancreatic islets incubated with CS-UCNP@PMVEMA-LGL nanoparticles after excitation at 808 nm (blue) and 980 nm (red).

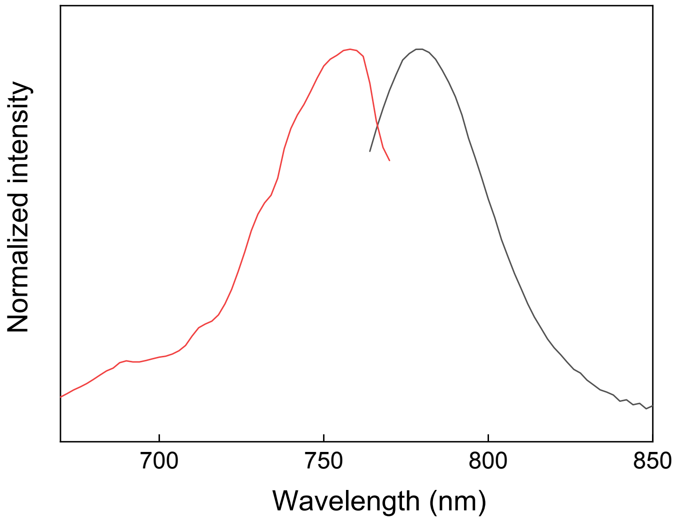

Figure S7. Photoluminescence excitation (red; λ_{em} = 780 nm) and emission (black; λ_{ex} = 758 nm) spectra of CS-UCNP@PMVEMA-LGL-Flamma® particles.

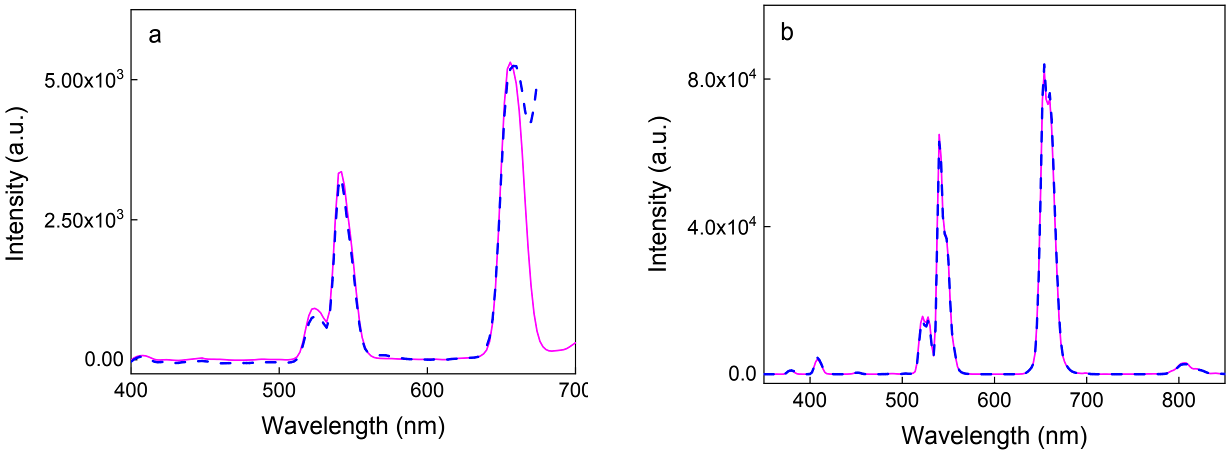

Figure S8. Emission spectra of upconversion photoluminescence of CS-UCNP@PMVEMA-LGL (pink solid) and CS-UCNP@PMVEMA-LGL-Flamma® particles (blue dash) in water (1 mg/ml) excited at (a) 808 and (b) 980 nm with laser power densities of 3 and 2.11 W/cm^2, respectively.

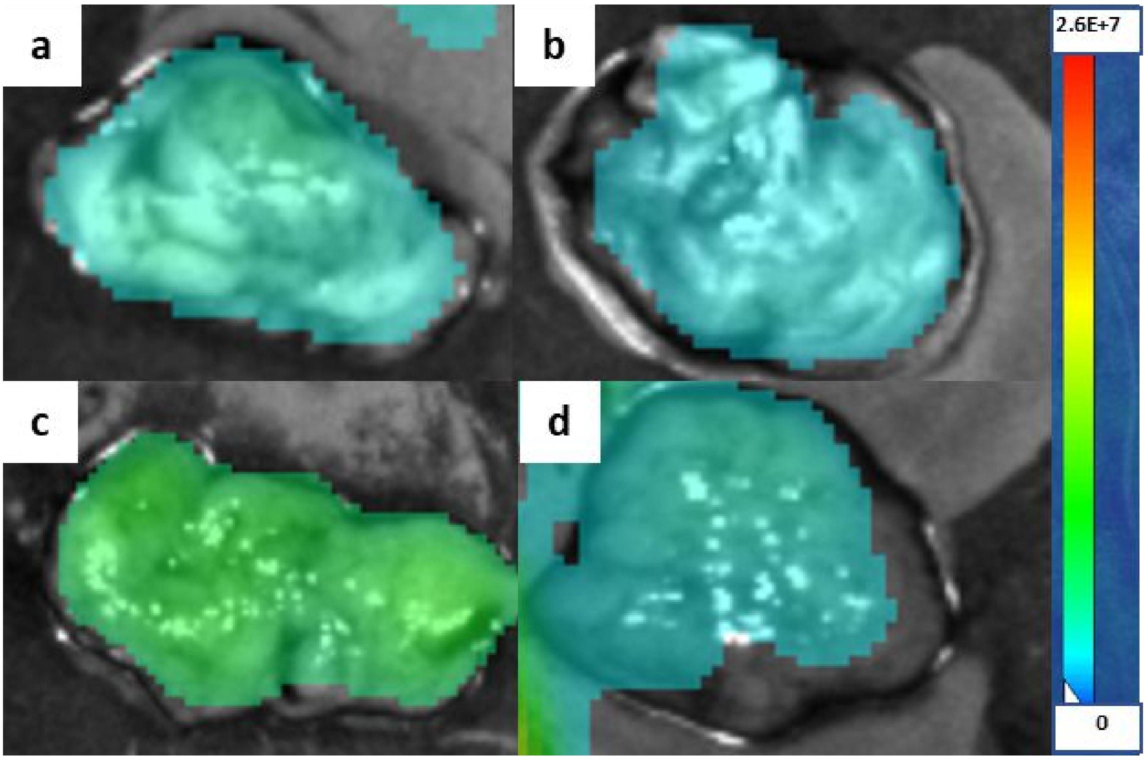

Figure S9. Fluorescence imaging and corresponding mean fluorescence intensity of CS-UCNP@PMVEMA-LGL-Flamma® nanoparticles accumulated in the pancreas (a, b) 3 and (c, d) 24 h after (a, c) intramuscular or (b, d) intravenous administration. Luminescence units are in radians (photons/s/cm^2). (a, b) $2.6 \cdot 10^7$, (c) $6.3 \cdot 10^7$, and (d) $3.4 \cdot 10^7$ rad.

References

1. Kostiv, U.; Engstová, H.; Krajnik, B.; Šlouf, M.; Proks, V.; Podhorodecki, A.; Ježek, P.; Horák, D. Monodisperse core-shell $NaYF_4:Yb^{3+}/Er^{3+}@NaYF_4:Nd^{3+}$-PEG-GGGRGDSGGGY-$NH_2$ nanoparticles excitable at 808 and 980 nm: Design, surface engineering, and application in life sciences. *Front. Chem.* **2020**, *8*, 497. doi: 10.3389/fchem.2020.00497

2. Andrews, K. W.; Dyson, D. J.; Keown, S. R. *Interpretation of Electron Diffraction Patterns*, 2nd ed.; Springer, New York, 1967. doi: 10.1007/978-1-4899-6475-5
